# Liver‐Secreted Extracellular Vesicles Promote Cirrhosis‐Associated Skeletal Muscle Injury Through mtDNA‐cGAS/STING Axis

**DOI:** 10.1002/advs.202410439

**Published:** 2025-01-13

**Authors:** Xiaoli Fan, Yunke Peng, Bo Li, Xiaoze Wang, Yifeng Liu, Yi Shen, Guofeng Liu, Yanyi Zheng, Qiaoyu Deng, Jingping Liu, Li Yang

**Affiliations:** ^1^ Department of Gastroenterology and Hepatology and Laboratory of Gastrointestinal Cancer and Liver Disease West China Hospital Sichuan University Chengdu 610041 China; ^2^ Department of Radiology West China Hospital Sichuan University Chengdu 610041 China; ^3^ NHC Key Laboratory of Transplant Engineering and Immunology Center for Disease‐related Molecular Network West China Hospital of Sichuan University Chengdu 610041 China

**Keywords:** cGAS‐STING, extracellular vesicles, liver fibrosis, mtDNA, skeletal muscle atrophy

## Abstract

Skeletal muscle atrophy (sarcopenia) is a serious complication of liver cirrhosis, and chronic muscle inflammation plays a pivotal role in its pathologenesis. However, the detailed mechanism through which injured liver tissues mediate skeletal muscle inflammatory injury remains elusive. Here, it is reported that injured hepatocytes might secrete mtDNA‐enriched extracellular vesicles (EVs) to trigger skeletal muscle inflammation by activating the cGAS‐STING pathway. Briefly, injured liver secreted increased amounts of EVs into circulation, which are then engulfed primarily by macrophages in skeletal muscle and subsequently induce cGAS‐STING signaling and its‐mediated inflammatory response in muscles. In contrast, suppression of hepatic EV secretion or STING signaling significantly alleviated cirrhosis‐induced skeletal muscle inflammation and muscle atrophy in vivo. Circulating EVs from cirrhotic patients showed higher levels of mtDNA, and the levels of EV‐mtDNA positively correlated with the severity of liver injury. In injured hepatocytes, mitochondrial damage promoted the release of cytosolic mtDNA and the subsequent secretion of mtDNA‐enriched EVs. This study reveals that injured hepatocyte‐derived EVs induce skeletal muscle inflammation via the mtDNA‒STING axis, while targeted blockade of liver EV secretion or STING signaling represents a potential therapeutic approach for preventing cirrhosis‐associated skeletal muscle atrophy.

## Graphical abstract

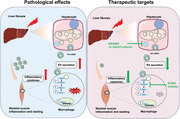

## 1 Introduction

Cirrhosis represents the advanced stage of various chronic liver diseases and affects ≈100 million patients worldwide.^[^ ^1^ ^]^ Moreover, progressive cirrhosis is strongly associated with multiple complications, such as sarcopenia, osteoporosis, and frailty.^[^ ^2^ ^]^ Sarcopenia, characterized by progressive skeletal muscle atrophy, is highly prevalent among individuals with cirrhosis, with an incidence ranging from 23% to 70%, and is also a significant independent risk factor for mortality in patients with cirrhosis.^[^ ^3^ ^]^ Although some potential interventions for preventing sarcopenia, such as nutritional supplements, exercise, and small molecular drug treatments, have been proposed, their clinical efficacy is still unsatisfied due to the complicated pathology of sarcopenia.^[^ ^4^ ^]^ The pathogenesis of cirrhosis‐associated sarcopenia is complex, and many factors, such as hyperammonemia, oxidative stress, and an abnormal immune response, are involved.^[^ ^5^, ^6^ ^]^ Chronic muscle inflammation, such as increased levels of inflammatory cytokines in mouse muscle tissues (e.g., TNF‐α and IL‐6), has been found in the cirrhotic state, and it may promote skeletal muscle injury via diverse mechanisms.^[^ ^7^, ^8^ ^]^ However, the detailed mechanism by which injured liver tissues mediate skeletal muscle inflammation in patients with cirrhosis is still poorly understood.

In recent years, mounting evidence has suggested that the interactions between the liver and extrahepatic organs/tissues (e.g., heart, gut, and bone) and such organ‒organ crosstalk play critical roles in the pathogenesis of multiple diseases, such as hepatic cardiomyopathy and osteoporosis.^[^ ^9^, ^10^ ^]^ Extracellular vehicles (EVs) are small bilayer vesicles secreted by tissue cells that can enter the circulation system and reach distant tissues after being released into the extracellular space.^[^ ^11^ ^]^ Thus, EVs play crucial roles in organ‐to‐organ communication by transferring various types of signaling molecules, such as protein lipids, nucleic acids, and metabolites.^[^ ^12^, ^13^ ^]^ For example, healthy liver tissue‐derived EVs exert endocrine signaling on peripheral tissues to regulate postprandial blood glucose levels,^[^ ^9^ ^]^ whereas steatotic liver tissue‐derived EVs aggravate atherosclerosis by inhibiting ATP binding cassette subfamily A member 1‐mediated cholesterol efflux via miR‐30a‐3p.^[^ ^14^ ^]^ However, whether cirrhosis‐derived EVs are major factors affecting the hepatic muscle axis, as well as the possible effects of hepatic EVs on regulating the liver‒muscle axis during skeletal muscle atrophy development, remains unclear.

In this study, for the first time, we present experimental and clinical evidence demonstrating that hepatic EV secretion assumes a critical role in the pathology of cirrhosis‐associated skeletal muscle atrophy. In brief, we disclose that, during cirrhosis, EVs derived from injured hepatocytes containing abundant damaged mitochondrial contents (such as mtDNA) can promote skeletal muscle inflammation and atrophy by activating the cGAS‒STING pathways in muscle macrophages. Mitochondrial injury in hepatocytes leads to cytosolic mtDNA leakage and the subsequent release of excessive mtDNA‐enriched EVs, and these EVs further exert proinflammatory effects in skeletal muscles. Conversely, inhibition of hepatic EV secretion through a pharmacological tool (N‐SMase inhibitor, GW4869) or a genetic tool (liver‐specific *Rab27a* deletion) significantly mitigates skeletal muscle injury in cirrhotic mice. This study reveals the crucial role of hepatic EVs in promoting skeletal muscle inflammation injury during cirrhosis and highlights that targeted inhibition of hepatic EV secretion or STING signaling constitutes a potential therapeutic approach for preventing cirrhosis‐associated muscle atrophy.

## 2 Results

### 2.1 Elevated Skeletal Muscle Atrophy and Inflammation in Cirrhotic Conditions

**Figure 1:**
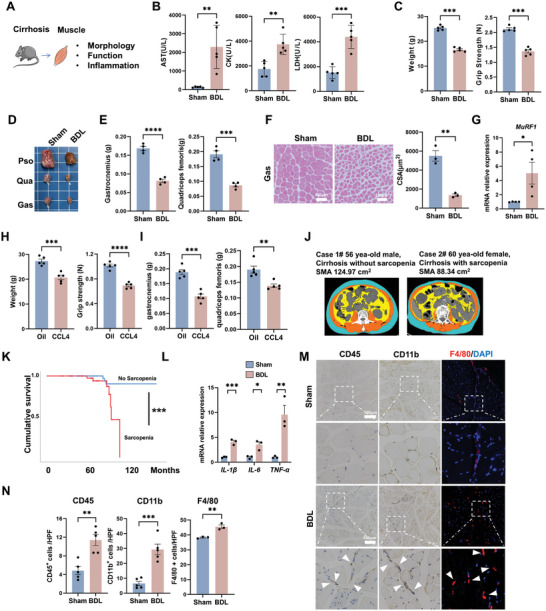
Elevated skeletal muscle atrophy and inflammation in cirrhotic conditions. A) Experimental scheme. Mice that underwent BDL were sacrificed after 2 weeks, after which the muscles were collected. B) Serum levels of AST, CK, and LDH in sham and BDL mice (*n* = 5). C) Body weight and grip strength of sham and BDL mice (*n* = 5). D) Representative images of dissected psoas major, quadriceps femoris, and gastrocnemius muscles. E) Weights of the quadriceps femoris and gastrocnemius muscles in sham and BDL mice (*n* = 4). F) Representative images of HE‐stained gastrocnemius muscle cross‐sections and quantification of the mean cross‐sectional area (*n* = 3). G) Expression levels of *MuRF1* measured by qPCR (*n* = 4). H) Body weight and grip strength of oil‐treated and CCL_4_‐treated mice (*n* = 5). I) Weights of the quadriceps femoris and gastrocnemius muscles in oil‐treated and CCL_4_‐treated mice (*n* = 5). J) Representative image of the third lumbar vertebra (L3) cross‐sectional CT image, which shows various skeletal muscles, visceral adipose tissue, and skeletal muscle between cirrhosis patients with sarcopenia and cirrhosis patients without sarcopenia. Orange, skeletal muscle area; blue, subcutaneous adipose tissue area; yellow, visceral adipose tissue area. K) Cumulative survival between the two groups. *p* < 0.005. L) Expression levels of *IL‐1β*, *IL‐6*, and *TNF‐α* measured by qPCR (*n* = 3). M,N) Representative images of IHC staining (CD45, CD11b) and the numbers of CD45‐positive and CD11b‐positive cells (scale bar = 100 µm, *n* = 5). Representative images of F4/80 IF staining (scale bar = 100 µm, *n* = 5) and F4/80‐positive cells (scale bar = 100 µm, *n* = 3). The white arrows indicate positively stained cells. The data are presented as the mean ± s.e.m. *p < 0.05, **p < 0.01, ***p < 0.001, ****p < 0.0001; two‐tailed unpaired *t*‐test was used for comparison.

The changes in skeletal muscle function were initially evaluated in two types of mouse cirrhosis models, namely bile duct ligation (BDL) and carbon tetrachloride (CCL_4_) injection (**Figure** **1**A). Both the BDL and CCL_4_ mice demonstrated liver inflammation and severe hepatic fibrosis (Figure S1A,B, Supporting Information). In contrast to the sham group, the BDL group exhibited increased systemic muscular injury and muscle atrophy, as indicated by elevated levels of skeletal muscle markers, namely, serum aspartate aminotransferase (AST), creatine kinase (CK) and lactic dehydrogenase (LDH) (Figure 1B),^[^ ^15^ ^]^ as well as decreased body weight, muscle tissue weight, grip strength and muscle fiber cross‐sectional area (CSA) at 2 weeks after surgery (Figure 1C–F). MuRF1, a major regulatory protein that accelerates muscle atrophy by mediating protein polyubiquitination and proteolysis, was also upregulated in muscle tissues from the BDL group compared to those from the sham group (Figure 1G; Figure S2A, Supporting Information).

In addition, lower body weight, grip strength, and muscle tissue weight were noted in the CCL_4_‐induced cirrhosis group compared to the oil group (Figure 1H,I). Similarly, a significant increase in MuRF1 expression was observed in the muscle tissue of mice with CCL4‐induced cirrhosis (Figure S2B, Supporting Information). Based on the previously reported clinical guidelines,^[^ ^16^ ^]^ the existing CT images of cirrhotic patients (*n* = 137) from the electronic medical records system at our institution were collected and analyzed. The development of skeletal muscle atrophy can be observed in cirrhotic patients, and Figure 1J presents representative abdominal CT scans at the L3 level of 2 cirrhotic patients with or without sarcopenia from a cohort of 137 cirrhotic patients in West China Hospital. Consistent with previous reports,^[^ ^5^, ^6^ ^]^ the results of Kaplan–Meier analysis indicated that the patients with cirrhosis with sarcopenia demonstrated a significantly poorer liver transplantation‐free survival rate compared to patients with cirrhosis but without sarcopenia (Figure 1K), suggesting an increased incidence of mortality in cirrhotic patients with clinically concomitant sarcopenia. Hence, in‐depth clarification of the underlying mechanism of liver cirrhosis‐related muscle injury and the development of targeted therapeutic interventions are urgently required.

Moreover, chronic inflammation is correlated with the development of muscle atrophy during cirrhosis. Compared with those of the sham group, the skeletal muscle tissues of the BDL group exhibited increased levels of proinflammatory factors (e.g., *IL‐β, IL‐6, and TNF‐α*) and infiltration of innate immune cells, including CD45^+^ leucocytes, CD11b^+^ myeloid cells and F4/80^+^ macrophages (Mφs), in the gastrocnemius muscle (Figure 1L–N). Nevertheless, there was no significant difference in the number of CD3^+^ T cells, CD4^+^ T cells, or CD8^+^ T cells between the groups (Figure S3, Supporting Information). Collectively, these results indicate increased skeletal muscle atrophy and inflammation in cirrhosis patients and that abnormally activated innate immune cells (e.g., macrophages) might participate in cirrhosis‐induced skeletal muscle inflammation.

### 2.2 Injured Liver‐Derived EVs Promote Skeletal Muscle Inflammatory Injury In Vivo

**Figure 2:**
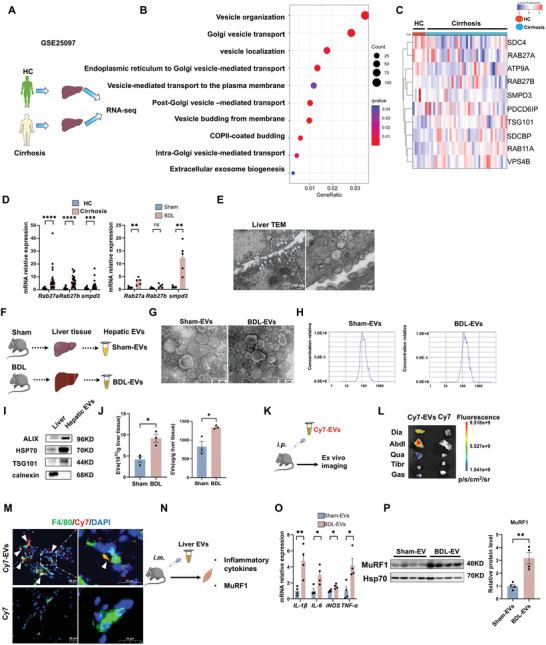
Injured liver‐derived EVs promote skeletal muscle inflammatory injury in vivo. A) Experimental scheme of the data extraction process for GSE25097. B) GO enrichment analysis of DEGs in the GSE25097 dataset (FC > 1.5 and p‐adjusted <0.05). C) Heatmaps of the changes in EV‐related genes. D) The expression levels of *Rab27a*, *Rab27b*, and *smpd3* in the human liver were measured via qPCR (*n* = 14/22) expression and levels of Rab27a, Rab27b, smpd3 measured by qPCR in Sham and BDL mice (*n* = 5). E) Representative TEM images of hepatic EVs (scale bar = 200 nm). The white arrows indicate the secretion of EVs. F) Experimental scheme. Extraction of hepatic EVs. G) Representative TEM images of Sham‐EVs and BDL‐EVs (scale bar = 200 nm). H) Size distributions of EVs measured by NTA. I) Western blot analysis of EV markers (ALIX, HSP70, TSG101, and calnexin). J) Two indicators for evaluating EV generating capacity (n = 3). (K‐L) Experimental scheme. Detection of the biodistribution of Cy7‐labeled EVs in vivo. M) Representative images showing the colocalization of F4/80 with Cy7‐labeled EVs. Mice that received equal amounts of free Cy7 solution were used as dye controls (scale bar = 20 µm). N) Experimental scheme. Intramuscular injection of Sham/BDL‐EV in mice. O) Expression levels of *IL‐1β*, *IL*‐6*, TNF‐α*, and *iNOS* measured by qPCR (*n* = 4). P) Representative blots and quantified MuRF1 protein expression data (*n* = 4). The data are presented as the mean ± s.e.m. *p < 0.05, **p < 0.01; two‐tailed unpaired *t*‐test was used for comparison.

To acquire insights into alterations in EV secretion‐related pathways in the fibrotic liver, RNA‐seq data (GSE25097) of liver tissues from 40 cirrhotic patients and 6 healthy controls (HCs) were analyzed (**Figure** **2**A; Figure S4A, Supporting Information). GO pathway enrichment analysis disclosed that numerous upregulated differentially expressed genes (DEGs), such as *Rab11A*, *TSG101*, and *VPS4B*, were enriched in pathways related to EV biogenesis, such as vesicle organization, localization, and transport (Figure 2B,C; Figure S4A, Supporting Information). The biogenesis processes of EVs are intricate and can be affected by multiple pathways, such as soluble N‐ethylmaleimide‐sensitive factor‐activating protein receptor (nSMase), neutral sphingomyelinase (Smpd3), and Rab GTPases (e.g., *Rab27a/Rab27b, Rab11)*, and their expression may vary under different conditions despite an overall elevated EV secretion.^[^ ^17^ ^]^ For example, upregulated liver Rab27a expression has been found in various chronic liver diseases, such as nonalcoholic fatty liver disease and CCL_4_‐induced cirrhosis models.^[^ ^18^, ^19^ ^]^ Correspondingly, we also identified increased gene expression levels of *Rab27a*/*Rab27b* (Figure 2D; Figure S4C,D, Supporting Information) and *smpd3* (Figure 2D; Figure S4C, Supporting Information) in the livers of our cirrhotic patient cohort and in BDL and CCL_4_‐induced cirrhosis mice, while the expression of other Rab GTPases (e.g., *Rab11a, Rab7a*, and *Rab35*) showed no significant difference in the livers of our cirrhotic patient cohort (Figure S4B, Supporting Information). TEM images further verified the existence of numerous secreted EVs (≈30–200 nm in diameter) in the hepatic sinusoid of the liver tissues of the BDL mice (Figure 2E). Additionally, EVs from the liver tissues of sham (Sham‐EVs) or BDL mice (BDL‐EVs) were isolated and characterized (Figure 2F). Sham‐EVs and BDL‐EVs displayed typical bilayer lipid membrane microstructures (Figure 2G), and their average sizes were comparable (≈130 nm) (Figure 2H). Both types of EVs were positive for marker proteins (Alix, TSG101, and HSP70) and negative for the expression of calnexin (an endoplasmic reticulum protein) (Figure 2I). The EV yield of the BDL group was significantly higher than that of the sham group (Figure 2J), suggesting enhanced hepatic EV secretion during cirrhosis.

Next, we ascertained whether liver‐secreted EVs could reach muscle tissues via the circulation in vivo. Since EVs are crucial mediators of organ‐to‐organ communication in physiological or pathological conditions. Hepatic EVs isolated from BDL mice were labeled with fluorescent Cy7 dye (Cy7‐EVs) and then systemically injected (*i.p*.) into BDL mice. In accordance with previous reports,^[^ ^20^ ^]^ positive Cy7‐EVs signals were observed in multiple skeletal muscle tissues (diaphragm, abdominal, quadriceps, tibialis anterior, quadriceps, and gastrocnemius) at 1‐h post‐injection (Figure 2K,L). Furthermore, numerous Cy7‐EVs were engulfed by F4/80^+^ macrophages in gastrocnemius tissues (Figure 2M). To evaluate the pathological role of hepatic EVs in skeletal muscle injury, Sham‐EVs or BDL‐EVs were injected into the gastrocnemius muscle of normal mice (1 × 10^10^ particles per mouse, three times per week) (Figure 2N). Compared with Sham‐EVs treatment, BDL‐EVs treatment for 1 week increased the expression levels of proinflammatory factors (such as IL‐1β and IL‐6) and MuRF1 in the skeletal muscle tissues of the mice (Figure 2O,P). Collectively, these results suggest that hepatic EV secretion in cirrhosis patients is higher and that injured liver‐derived EVs can facilitate skeletal muscle inflammatory injury in vivo.

### 2.3 Inhibiting Hepatic EV Secretion Reduces Skeletal Muscle Inflammatory Injury in Cirrhotic Mice

**Figure 3:**
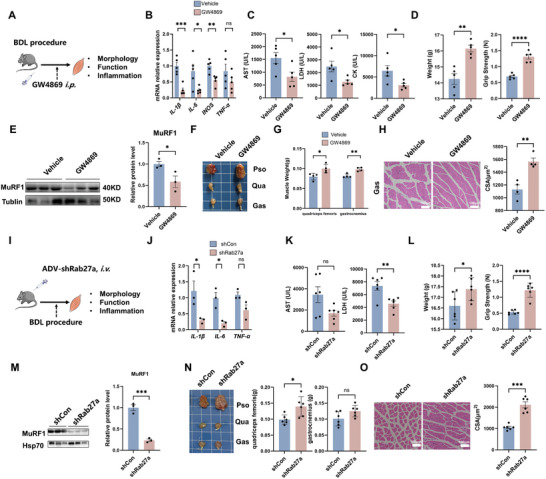
Inhibiting hepatic EV secretion reduces skeletal muscle inflammatory injury during cirrhosis. A) Experimental scheme. After the BDL operation, the mice were intraperitoneally injected with GW4869 or vehicle. After 2 weeks, the mice were sacrificed, and blood and muscles were collected. B) Expression levels of *IL‐1β*, *IL‐6*, *TNF‐α*, and *iNOS* measured by qPCR (*n* = 5). C) Serum levels of AST, CK, and LDH in the vehicle and GW4869 groups (*n* = 5). D) Body weight and grip strength of the vehicle and GW4869 groups (*n* = 5). E) Representative blots and quantified MuRF1 protein expression data (*n* = 3). F) Representative images of dissected psoas major, quadriceps femoris, and gastrocnemius muscles. G) Weights of the quadriceps femoris and gastrocnemius muscles in the vehicle and GW4869 groups (*n* = 4). H) Representative images of HE‐stained cross‐sections of the gastrocnemius muscle and quantification of the mean cross‐sectional area (*n* = 4). I) Experimental scheme. Two days after the BDL operation, the mice were injected with *shRab27a* or sh*Con*. After 2 weeks, the mice were sacrificed, and the muscles were collected. J) The expression levels of *IL‐1β*, *IL‐6*, and *TNF‐α* were measured via qPCR (*n* = 3). K) Serum levels of AST and LDH in the sh*Rab27*a and sh*Con* groups (*n* = 6). L) Body weight and grip strength of the sh*Rab27a* and sh*Con* groups (*n* = 6). M) Representative blots and quantified data showing the expression of the MuRF1 protein (*n* = 3). N) Representative images of dissected psoas major, quadriceps femoris, and gastrocnemius muscles. Body weight and grip strength of the shRab27a and shCon groups (*n* = 6). O) Representative images of HE‐stained gastrocnemius muscle cross‐sections and quantification of the mean cross‐sectional area (*n* = 6). The data are presented as the mean ± s.e.m. ^ns^ *p* ≥ 0.05, *p < 0.05, **p < 0.01, ***p < 0.001, ****p < 0.0001; a two‐tailed unpaired *t*‐test was used for comparison.

To validate the role of injured liver‐derived EVs in inducing skeletal muscle injury, EV secretion in liver tissues in vivo was inhibited via a specific inhibitor (GW4869, an N‐SMase/smpd3 inhibitor) or genetic tools. BDL mice were intraperitoneally (*i.p*.) administered with GW4869 (three times per week) for 2 weeks (**Figure** **3**A). GW4869 treatment decreased EV production (determined by EV particle numbers or EV protein amounts) in injured liver tissues from BDL mice (Figure S5A, Supporting Information) and slightly diminished EV secretion in several other normal organs (e.g., heart, lung, and kidney). As our data indicated that other normal organs seem to secrete fewer EVs (≈10^9^–10^11^ particles g^−1^ tissue) than injured livers (≈10^12^ particles g^−1^ tissue) in BDL mice did, we hypothesized that systemic GW4869 treatment might predominantly affect hepatic EV secretion in a cirrhosis model. As depicted in Figure S5B,C (Supporting Information), GW4869 treatment partially mitigated hepatic fibrosis, as evidenced by liver α‐SMA expression and collagen formation. Moreover, GW4869 treatment significantly reduced the gene expression of proinflammatory factors (e.g., *IL‐β, IL‐6, and iNOS*) in the skeletal muscle tissues of the BDL mice (Figure 3B). Consequently, compared with those treated with BDL alone, BDL mice treated with GW4869 exhibited a lower degree of skeletal muscle injury and atrophy, as manifested by lower serum levels (AST, CK and LDH) and muscle MuRF1 expression and greater body weight, muscle weight, grip strength, and muscle CSA (Figure 3C–H), suggesting that inhibition of hepatic EV secretion is an effective means to alleviate muscle injury and atrophy in cirrhosis.

Rab27a and Rab27b, belonging to the Rab family of small GTPases, and play crucial and diverse roles in regulating EV biogenesis. In this study, *Rab27a* expression was upregulated in the livers of patients with cirrhosis and in BDL mice, and its expression tended to be greater than that of *Rab27b* (Figure 2D). It has been reported that *Rab27a*‐knockdown might exert a more profound inhibitory effect on exosome secretion compared to *Rab27b*‐knockdown,^[^ ^21^ ^]^ which has been widely utilized to reduce hepatic EV release in many previous studies.^[^ ^14^, ^18^ ^]^ Based on these reports, we also specifically silenced *Rab27a* expression in hepatocytes of mouse liver tissue using an adenoviral vector (AdV) with a U6 promoter (Figure 3I; Figure S6A, Supporting Information). After the injection of EGFP‐tagged AdV via the tail vein, enhanced green fluorescent protein (EGFP)‐expressing hepatocytes and downregulated *Rab27a* expression were observed in the liver tissues of the BDL mice (Figure S6B,C, Supporting Information), suggesting the effective inhibition of *Rab27a*‐mediated EV secretion in hepatocytes. Additionally, hepatocyte‐specific *Rab27a* silencing significantly mitigated skeletal muscle inflammatory injury in BDL mice, as evidenced by reductions in the levels of serum indicators (AST, CK, and LDH) and muscle proinflammatory factors and increases in body weight, muscle weight, grip strength, and muscle CSA, compared to those in the BDL alone group (Figure 3J–O). Collectively, our results suggest that inhibiting hepatic EV secretion can alleviate skeletal muscle inflammation and wasting in cirrhotic mice.

### 2.4 Injured Liver‐Derived EVs Induce Skeletal Muscle Inflammation by Activating cGAS‐STING Signaling

**Figure 4:**
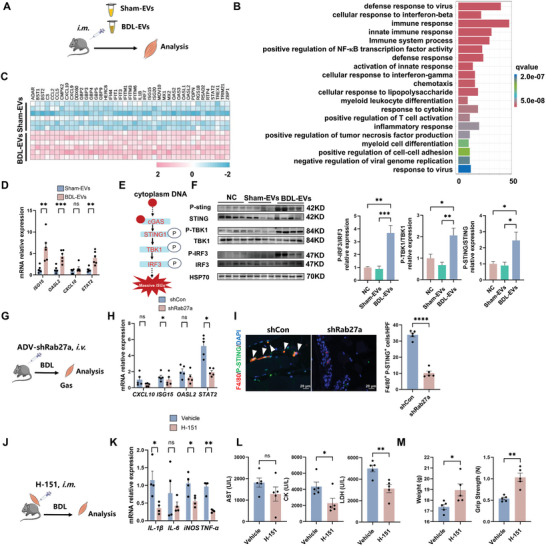
Injured liver‐derived EVs induce muscle inflammation by activating cGAS‐STING in vivo. A) Experimental scheme. Intramuscular injection of Sham/BDL‐EV in mice. B) GO enrichment analysis of DEGs (FC > 1.5, top 20). C) Heatmap showing the different gene expression profiles in the different groups. D) Expression levels of *ISG15*, *OASL2*, *CXCL10*, and *STAT2* measured by qPCR (*n* = 6). E) Schematic map of the cGAS‐STING signaling pathway. F) Representative blots and quantified data showing the protein expression of STING, P‐STING, TBK1, P‐TBK1, IRF3, and P‐IRF3 (*n* = 4). G) Experimental scheme. Two days after the BDL operation, the mice were injected with sh*Rab27a* or sh*Con*. H) Expression levels of *ISG15*, *OASL2*, *CXCL10*, and *STAT2* measured by qPCR (*n* = 5). I) Representative images showing the colocalization of F4/80 with P‐STING in muscle and quantification of the number of F4/80‐ and P‐STING‐positive cells (scale bar = 20 µm, *n* = 5). Arrows indicate F4/80 and P‐STING dual‐positive cells. J) Experimental scheme. After the BDL operation, the mice were intramuscularly injected with H‐151 or vehicle. K) Expression levels of *IL‐1β*, *IL‐6*, *TNF‐α*, and *iNOS* measured by qPCR (*n* = 4). L) Serum levels of AST, CK, and LDH in vehicle‐treated or H‐151‐treated mice (*n* = 5). M) Body weight and grip strength of vehicle‐treated or H‐151‐treated mice (*n* = 5). The data are presented as the mean ± s.e.m. ^ns^ *p* ≥ 0.05, *p < 0.05, **p < 0.01, ***p < 0.001; two‐tailed unpaired *t*‐test for comparison; one‐way ANOVA for multiple comparisons with Tukey's test for post hoc corrections.

To disclose the detailed mechanism of injured liver‐derived EVs in skeletal muscle injury, the global gene expression of muscle tissues after Sham‐EVs or BDL‐EVs treatment was characterized via RNA‐seq (**Figure** **4**A). Heatmaps and PCA plots demonstrated distinct gene expression patterns between the groups (Figure S7A,B, Supporting Information). The differentially expressed genes (DEGs) affected by the BDL‐EVs were predominantly enriched mainly in pathways related to the innate immune response, such as the defense response to viruses, the cellular response to interferon‐beta, the immune response, and the cellular response to interferon‐gamma (the top 20 pathways) (Figure 4B). The upregulated DEGs in the BDL‐EVs group were implicated in multiple proinflammatory pathways, such as stimulator of interferon genes (ISGs) (*ISG15, CXCL10, OASL2, and STAT2*) and chemotaxis (*CCL2, CCL5, CXCL9, and RGS18*) pathways (Figure 4C). Further qPCR assays verified the upregulation of ISGs, such as *ISG15, OASL2, CXCL10*, and *STAT2*, in the muscle tissues of the BDL‐EVs group (Figure 4D).

The cGAS‐STING pathway plays a crucial role in regulating the innate immune response by inducing the ISG/IFN signaling pathway and the expression of numerous proinflammatory cytokines (e.g., TNF‐α and IL‐6).^[^ ^22^ ^]^ The protein expression of key cGAS‐STING signaling molecules in muscles was determined (Figure 4E), and this pathway was activated in muscle tissues of the BDL‐EVs group (Figure 4F). Elevated ISG expression was also identified in the muscle tissues of the BDL group (Figure S7C, Supporting Information), suggesting that cirrhosis‐derived EVs may promote muscle inflammation by activating the cGAS‐STING pathway. To validate these findings, we also evaluated the alterations in the cGAS–STING pathway in the muscle of the BDL mice with hepatocyte‐specific *Rab27a* depletion (Figure 4G). Inhibition of hepatic EV secretion decreased cGAS‐STING‐induced ISG (e.g., *ISG15* and *STAT2*) expression in the skeletal muscle tissues of the BDL mice (Figure 4H). Hepatic *Rab27a* silencing also reduced the levels of p‐STING protein in F4/80^+^ macrophages in muscle tissues of BDL mice (Figure 4I), suggesting that hepatic EVs might activate the cGAS–STING pathway in macrophages in skeletal muscle tissues. Compared with Sham‐EVs group, macrophages primed with BDL‐EVs displayed increased protein levels of STING activation (Figure 4F), while hepatic EVs slightly increased p‐STING expression in myotubes (Figure S9G, Supporting Information), suggesting a relative weaker response to such EVs compared to macrophages.

Next, we ascertained whether STING inhibition could alleviate muscle injury in cirrhosis by intramuscular injection of a specific STING inhibitor (H‐151) (Figure 4J), as this inhibitor was demonstrated to suppress ISGs and cytokine expression in vivo.^[^ ^23^ ^]^ In BDL mice, H‐151 treatment suppressed the expression of ISGs (*CXCL10, ISG15*, and *STAT2*), proinflammatory factors, and muscle injury biomarkers (Figure S7D, Supporting Information; Figure 4K,L) and enhanced body weight and grip strength (Figure 4M). The mechanism of hepatic EVs‐induced muscle inflammation is intricate, and multiple pathways might be implicated. In addition to the STING pathway, the RNA‐seq results of muscles indicated that hepatic EVs also influenced other proinflammatory pathways, such as the NF‐κB pathway and chemotaxis (Figure 4B). Furthermore, treatment with an NF‐κB inhibitor (Bay 11–7085) or a CCR2/CCR5 inhibitor (cenicriviroc, CVC) decreased the levels of muscle injury indicators, including AST and CK (Figure S8B,E, Supporting Information), respectively, while the NF‐κB inhibitor also improved body weight and grip strength (Figure S8C, Supporting Information). However, CCR2/CCR5 inhibitor treatment had no significant effect on body weight or grip strength in the BDL mice (Figure S8F, Supporting Information). It has been reported that cGAS‐STING signaling can also induce the NF‐κB signaling pathway via TBK1 phosphorylation.^[^ ^24^ ^]^ These results suggest that multiple pathways, such as the STING and NF‐κB pathways, can be triggered by hepatic EVs and that these pathways might form a complex network that cooperates jointly to regulate the development of cirrhosis‐associated skeletal muscle injury. Collectively, our results suggest a crucial role of the hepatic EVs‐induced cGAS–STING pathway in promoting muscle injury during cirrhosis.

### 2.5 Injured Liver‐Derived EVs Activate the cGAS‒STING Pathway in Macrophages by Transferring mtDNA

**Figure 5:**
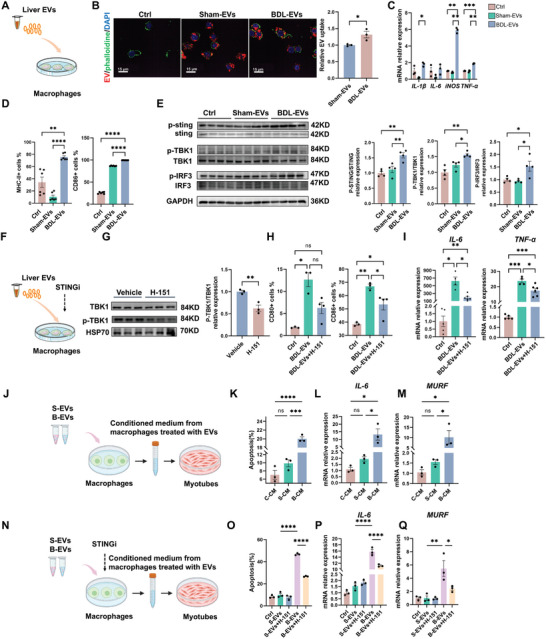
Injured liver‐derived EVs activate the cGAS‐STING pathway in macrophages in vitro. A) Experimental scheme. Detection of the effects of hepatic EVs on macrophages. B) Representative images of PKH26‐labeled EV (red) uptake in macrophages stained with FITC‐phalloidin (green) and DAPI (blue) and the relative uptake ratio of EVs in macrophages of different groups (scale bar = 15 µm, *n* = 3). C) Expression levels of *IL‐1β*, *IL‐6*, *TNF‐α*, and *iNOS* measured by qPCR (*n* = 3). D) FCM analysis of MHCII‐positive cells and CD86‐positive cells (*n* = 6). E) Representative blots and quantified data showing the expression of STING, P‐STING, TBK1, P‐TBK1, IRF3 and P‐IRF3 (*n* = 4). F) Experimental scheme. Detection of the effects of hepatic EVs on macrophages after treatment with H‐151. G) Representative blots and quantified data showing the protein expression of TBK1 and P‐TBK1 (*n* = 3). H) FCM analysis of CD80‐positive cells and CD86‐positive cells (*n* = 3–4). I) Expression levels of *IL‐6* and *TNF‐α* measured by qPCR (*n* = 4–6). J) Experimental scheme. Detection of the effects of CM from hepatic EV‐primed macrophages on myotubes. K) Determination of the cell apoptosis rate (%) of myotubes cultured with CM from hepatic EV‐primed macrophages (*n* = 3). L,M) Expression levels of *IL‐6* and *MURF* measured by qPCR (*n* = 3). N) Experimental scheme. Detection of the effects of CM from hepatic EV‐primed macrophages with or without a STING inhibitor on myotubes. O) Determination of apoptosis (%) in myotubes cultured with CM from hepatic EV‐primed macrophages with or without a STING inhibitor (*n* = 3). P,Q) Expression levels of *IL‐6* and *MURF* measured by qPCR (*n* = 3). The data are presented as the means ± s.e.m. ^ns^ *p* ≥ 0.05, *p < 0.05, **p < 0.01, ***p < 0.001, ****p < 0.0001; two‐tailed unpaired *t*‐test for comparison; one‐way ANOVA for multiple comparisons with Tukey's test for post hoc corrections.

Having disclosed the proinflammatory role of fibrotic liver‐derived EVs in vivo, we subsequently explored the detailed influence of such EVs on muscle‐resident cells and immune cells. Although Sham‐EVs and BDL‐EVs could be internalized up by C2C12‐derived myotubes in vitro (Figure S9A,B, Supporting Information), there was no significant difference in cell viability, cell apoptosis, proinflammatory factor expression, or MuRF1 expression between the EV groups and the control group (Figure S9C–F, Supporting Information). Intriguingly, native Mφs (M0) engulfed BDL‐EVs more effectively than Sham‐EVs(**Figure** **5**A,B). Compared with those in the Sham‐EVs group, Mφs treated with BDL‐EVs exhibited increased levels of M1‐like markers (MHC‐II and CD86), cytokines (e.g.*, IL‐1β, iNOS, and TNF‐α*) and STING activation (p‐STING, p‐TBK1, and p‐IRF3) (Figure 5C–E). Conversely, treatment with a STING inhibitor (H‐151) decreased the levels of p‐TBK1, an M1‐like marker (CD86) and cytokines in the BDL‐EVs‐primed Mφs (Figure 5F–I). These results suggest that hepatic EV‐induced skeletal muscle inflammation is likely attributed to activation of the cGAS‐STING pathway in muscle Mφs. C2C12‐derived myotubes were subsequently cocultured with CM from macrophages primed with BDL‐EVs or Sham‐EVs for 24 h with or without H‐151 treatment (Figure 5J–Q). Compared with those in the Sham‐EVs‐primed macrophage group, the myotubes in the BDL‐EVs‐primed macrophage group presented increased levels of cell apoptosis, *IL‐6* expression, and *MURF* expression (Figure 5J–M). In contrast, the ability of H‐151‐treated, BDL‐EVs‐primed macrophages to induce C2C12‐derived myotube damage was reduced (Figure 5N–Q). Collectively, our results indicate that BDL‐EVs promote the polarization of muscle macrophages toward a proinflammatory phenotype via activation of the cGAS‐STING pathway and subsequently trigger inflammation, apoptosis, and atrophy of muscle‐resident cells.

**Figure 6:**
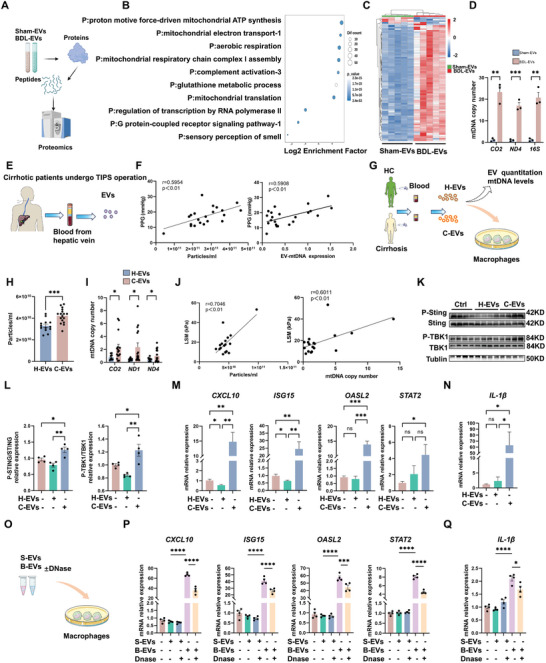
Injured liver‐derived EVs activate cGAS‐STING in macrophages by transferring mtDNA. A) Schematic illustrating the proteomics experiments. B) GO enrichment analysis of DEPs in different groups (FC > 1.5, top 10). C) Heatmap displaying mitochondria‐related variations in protein composition between the Sham‐EV group and the BDL‐EV group (*n* = 5). D) Relative mtDNA copy numbers of *CO_2_*, *ND4*, and *16S* in Sham‐EV and BDL‐EV (*n* = 3). E) Experimental scheme. The serum EVs were extracted from the hepatic vein collected during the TIPS operation. The EV‐mtDNA levels were determined by qPCR. F) Correlations between PPG and EV‐mtDNA levels and between PPG and EV‐particle levels. G) Experimental scheme. The effects of treatment with EVs from the blood of HCs or patients with cirrhosis on macrophages were detected. H) The number of EVs in the different groups (*n* = 13/17). I) Relative mtDNA copy number of *CO_2_*, *ND1*, and *ND4* (*n* = 9/16, respectively). J) Correlations between liver stiffness measurement (LSM) and EV‐mtDNA, LSM, and EV‐particle levels (N = 18). K,L) Representative blots and quantified data showing the protein expression of STING, P‐STING, TBK1, and P‐TBK1 (*n* = 4). M) Expression levels of *ISG15*, *OASL2*, *CXCL10*, and STAT2 measured by qPCR (*n* = 3–4). N) Expression levels of IL‐1β measured by qPCR (*n* = 3–4). O) Experimental scheme. Effects of EVs with or without mtDNA depletion on macrophages (*n* = 4). The depletion of mtDNA from EVs was performed via the DNase/Triton X‐100 method. P) Expression levels of *CXCL10*, *ISG15*, *OASL2* and *STAT2* measured by qPCR (*n* = 4). Q) Expression levels of *IL‐1β* measured by qPCR (*n* = 4). The data are presented as the mean ± s.e.m. *p < 0.05, ***p < 0.001, ****p < 0.0001; two‐tailed unpaired *t*‐test for comparison; one‐way ANOVA for multiple comparisons with Tukey's test for post hoc corrections.

To illuminate their proinflammatory mechanisms, the compositions of hepatic EVs were analyzed via LC‒MS/MS‐based proteomics (**Figure** **6**A). Differentially expressed proteins (DEPs, 625 downregulated and 1225 upregulated) between the BDL‐EVs group and the Sham‐EVs group were identified (Figure S10A,B, Supporting Information). The proteomics analysis of liver derived‐EVs revealed that BDL‐EVs contained more mitochondrial components (e.g, CO_2_, ND4) compared to Sham‐EVs (Figure 6B,C; Figure S10B, Supporting Information). The released mitochondrial damage‐associated molecular patterns (DAMPs), such as damaged mitochondrial proteins and mtDNA, are potent inducers of the innate immune response (e.g., cGAS‐STING) in diverse tissue types.^[^ ^25^, ^26^ ^]^ In addition, mtDNA is not solely presented; it usually binds to certain mitochondrial proteins (e.g., TFAM, PrimPol) to form mtDNA nucleoid.^[^ ^27^, ^28^ ^]^ We found that the levels of mtDNA copy number of BDL‐EVs were also higher than Sham‐EVs (Figure 6D). Thus, our results suggest that injured liver‐derived EVs may activate the cGAS‐STING pathway in muscle macrophages by transferring injured mitochondria‐derived contents, such as mtDNA.

To validate the findings from mouse cirrhosis models, changes in EV numbers and EV mtDNA levels in patients with cirrhosis were determined. Hepatic vein blood outflow from the hepatic parenchyma closely reflects metabolic changes in the liver; thus, EVs from the hepatic vein blood of patients with cirrhosis who underwent the TIPS procedure were isolated (Figure 6E). Increased EV numbers or EV mtDNA copy numbers were positively correlated with elevated levels of portal pressure (PPG, an indicator of portal hypertension) in patients with cirrhosis (Figure 6F). EVs from the peripheral blood of patients with cirrhosis or healthy controls (HCs) were also isolated (Figure 6G). Compared with the HCs, the patients with cirrhosis had a greater number of EVs and a greater number of EV mtDNA copies (Figure 6H,I). EV number and EV mtDNA content were also positively correlated with liver stiffness in patients with cirrhosis (Figure 6J). These results collectively suggest that EV secretion in injured livers is strongly associated with disease severity. Moreover, compared with blood EVs from the HCs, blood EVs isolated from the patients with cirrhosis exerted proinflammatory effects on macrophages, as indicated by increased p‐STING and p‐TBK1 protein levels, ISG expression, and proinflammatory factor levels (Figure 6K–N; Figure S11, Supporting Information). To verify the role of mtDNA carried by hepatic EVs in inducing the cGAS‐STING pathway, we have added a rescue experiment to deplete the mtDNA contents in EVs via DNase and 0.1% Triton X‐100 treatment, as previously reported.^[^ ^29^ ^]^ Compared with those in the BDL‐EVs‐treated group, in which exosomal mtDNA remained intact, EVs depleted of mtDNA (DNase/0.1% Triton X‐100) had reduced proinflammatory effects and lower *ISGs* and cytokines in macrophages (Figures 6O–Q; Figure S12, Supporting Information). Consequently, macrophages primed with mtDNA‐depleted EVs caused milder damage to myotubes (Figure S13, Supporting Information). These results suggest that injured hepatocyte‐derived EVs induce proinflammatory macrophages in muscles by activating the cGAS‒STING pathway and that this effect is at least partly dependent on the mtDNA content of such EVs.

### 2.6 Mitochondrial Injury Drives Hepatocytes to Release mtDNA‐Rich EVs to Induce Proinflammatory Macrophages

**Figure 7:**
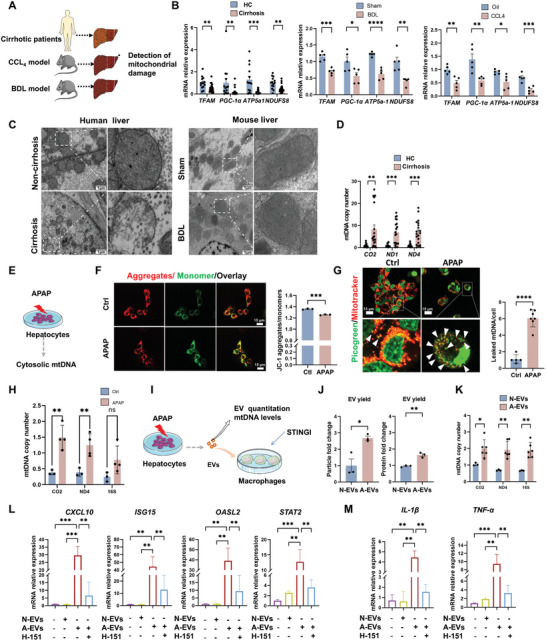
Mitochondrial injury drives hepatocytes to release mtDNA‐rich EVs to induce proinflammatory macrophages. A) Experimental scheme. Detection of mitochondrial damage in the livers of patients with cirrhosis and mouse models. B) Expression levels of *TFAM*, *PGC‐1α*, *ATP5a1*, and *NDUFS8* measured by qPCR in the livers of HCs and cirrhosis patients (*n* = 14/22). The expression levels of *TFAM*, *PGC‐1α*, *ATP5a1*, and *NDUFS8* in the livers of sham and BDL mice were measured by qPCR (*n* = 5). The expression levels of *TFAM, PGC‐1α, ATP5a1, and NDUFS8* in the livers of oil‐treated and CCl_4_‐treated mice were measured by qPCR (*n* = 5). C) Representative TEM image of human livers from patients with or without cirrhosis, and BDL mouse livers (scale bar = 1 µm). D) Relative mtDNA copy numbers of *CO_2_*, *ND1*, and *ND4* in the livers of HCs and patients with cirrhosis (*n* = 12/20). E) Experimental scheme. After hepatocytes were treated with APAP or vehicle, the MMP and the cytoplasmic mtDNA levels were determined. F) Representative images of JC‐1‐stained mitochondrial membrane potential (scale bar = 15 µm, *n* = 3). G) Representative images of the colocalization of dsDNA and mitochondria in hepatocytes. MitoTracker Red and PicoGreen were used for staining (scale bar = 15 µm, *n* = 5/7). The white arrows indicate cytoplasmic leaked mtDNA. H) Relative mtDNA copy number of *CO_2_*, *ND4*, and *16S* (*n* = 3/4). I) Experimental scheme. Detection of the effects of hepatocyte‐derived EVs on macrophages after treatment with H‐151. J) Two indicators for evaluating EV generating capacity (*n* = 3). K) Relative mtDNA copy number of *CO_2_*, *ND4*, and *16S* (*n* = 3/6). L) Expression levels of *ISG15*, *OASL2*, *CXCL10*, and *STAT2* measured by qPCR (*n* = 3–4). M) Expression levels of *IL‐1β* and *TNF‐α* measured by qPCR (*n* = 3–4). The data are presented as the mean ± s.e.m. ^ns^ *p* ≥ 0.05, *p < 0.05, **p < 0.01, ***p < 0.001, ****p < 0.0001; two‐tailed unpaired *t*‐test for comparison; one‐way ANOVA for multiple comparisons with Tukey's test for post hoc corrections.

Mitochondrial injury is mainly characterized by mtDNA damage, elevated oxidative stress, a reduced population of mitochondria, and impaired functionality. These lesions not only compromise cellular energy metabolism but also may trigger apoptosis and necrosis, thereby accelerating the progression of various diseases.^[^ ^30^ ^]^ In recent years, increasing evidence has indicated the crucial role of mitochondrial injury in the pathology of liver fibrosis.^[^ ^31^, ^32^ ^]^ For example, severe electron transport chain defects and decreased mitochondrial membrane potential have been identified in the livers of cirrhotic patients,^[^ ^32^ ^]^ and increased mitochondrial oxidative stress has been observed in cirrhotic liver conditions.^[^ ^33^, ^34^ ^]^ Previous studies have disclosed increased cytoplasmic mtDNA leakage in the context of mitochondrial injury and cytoplasmic mtDNA may also be released into the extracellular space, thereby inducing an innate immune response and inflammation in diverse tissue types.^[^ ^35^, ^36^, ^37^ ^]^ Having disclosed that injured liver‐derived EVs contain abundant mtDNA and can activate the cGAS‒STING pathway in muscle macrophages, we next determined whether mitochondrial injury is a crucial factor in the secretion of mtDNA‐enriched EVs in injured hepatocytes. We detected mitochondrial gene expression in liver specimens from patients with cirrhosis and mouse cirrhotic models, and the results indicated decreased expression levels of mitochondrial function‐related genes (*TFAM, PGC‐1α, ATP5a1*, *and NDUFS8*) in the liver tissues of both patients with cirrhosis and mice (**Figure** **7**A,B), reflecting severe hepatic mitochondrial injury during cirrhosis. Further TEM images of liver specimens revealed severe mitochondrial lesions, such as mitochondrial vesicular swelling, leakage, and pyknosis, in hepatocytes from patients with cirrhosis and BDL mice (Figure 7C). Moreover, the mtDNA content in the liver tissues of patients with cirrhosis and model mice was higher than that in the control group (Figure 7D; Figure S14, Supporting Information).

To validate the role of mitochondrial injury in promoting mtDNA‐rich EV release, mouse hepatocytes (AML12) were stimulated with acetaminophen (APAP) to induce mitochondrial injury as previously reported (Figure 7E).^[^ ^38^ ^]^ APAP treatment disrupted the mitochondrial membrane potential (MMP) of hepatocytes, as attested by increased levels of JC‐1 aggregates (yellow) (Figure 7F). Elevated levels of leaked dsDNA (green) in the cytoplasm and increased cytosolic mtDNA copy number (determined by qPCR) were detected in hepatocytes treated with APAP (Figure 7G,H), indicating augmented cytosolic mtDNA release during mitochondrial injury. Furthermore, M0 macrophages were co‐treated with normal control‐EVs (N‐EVs) or APAP‐EVs (A‐EVs) and a STING inhibitor (Figure 7I). Consequently, EVs from APAP‐treated hepatocytes exhibited greater EV yields and mtDNA contents than those from normal controls (Figure 7J,K), suggesting that mitochondrial injury triggers cytosolic mtDNA release in hepatocytes and subsequent mtDNA secretion into the extracellular space via EVs. A‐EVs treatment upregulated the gene expression of ISGs and cytokines in macrophages, whereas this effect was partially reversed after H‐151 treatment (Figure 7L,M; Figure S15, Supporting Information). Collectively, these results indicate that mitochondrial injury drives hepatocytes to release mtDNA‐enriched EVs to induce proinflammatory macrophages in muscle tissues.

## 3 Discussion

To date, although some pathological factors of cirrhosis‐related sarcopenia, such as metabolic disturbances, hyperammonemia, inflammation, and hormone alterations, have been reported,^[^ ^3^ ^]^ the detailed pathogenesis mechanism remains poorly understood. Emerging evidence has indicated that EV secretion plays a critical role in the crosstalk between the liver and certain extrahepatic organs/tissues (e.g., adipose tissue and the cardiovascular system) in both physiological or diseased states.^[^ ^14^, ^39^ ^]^ However, whether this process contributes to the development of cirrhosis‐related sarcopenia remains unclear. In this study, we demonstrated that cirrhotic liver‐derived EVs play a crucial role in the pathology of skeletal muscle inflammation and atrophy. During the progression of liver cirrhosis, mitochondrial injury in hepatocytes promotes the release of mtDNA‐enriched EVs, which further activate cGAS‐STING signaling in skeletal muscle macrophages by shedding mtDNA. Conversely, targeted inhibition of hepatic EV secretion or STING signaling can mitigate skeletal muscle inflammatory injury and atrophy in cirrhosis. To the best of our knowledge, this is the first report to provide evidence that hepatic EV release contributes to skeletal muscle atrophy in cirrhosis.

Previous studies have demonstrated that skeletal muscle becomes a major organ, alongside the liver, for ammonia uptake, while ureagenesis is impaired in hepatocytes in patients with cirrhosis. This impairment can promote cirrhosis‐induced sarcopenia via compromised mitochondrial respiration and reduced ATP synthesis in skeletal muscle cells.^[^ ^40^, ^41^ ^]^ In addition to hyperammonemia, liver‐derived cytokines or biologically active substances can mediate interorgan crosstalk in liver disease‐induced muscle atrophy.^[^ ^42^, ^43^ ^]^ For instance, TNF‐α derived from liver cirrhosis mediates skeletal muscle atrophy through the bloodstream,^[^ ^44^ ^]^ and TNF‐α treatment can lead to muscle atrophy through stimulating the expression of atrophy‐related E3 ubiquitin ligases in skeletal muscle cells (e.g., Atrogin1 and MuRF1).^[^ ^45^, ^46^ ^]^ Notably, chronic muscle inflammation is a pathological feature of diverse forms of sarcopenia.^[^ ^7^ ^]^ For example, aging‐associated muscle atrophy is accompanied by the upregulation of proinflammatory cytokines (e.g., *IL‐6* and *TNF‐α*) and the infiltrated immune cells (e.g., macrophages).^[^ ^47^ ^]^ EV secretion is a highly conserved biological process and serves as a potent mechanism for removing damaged materials from parent cells. Under stress conditions, hepatocytes may exhibit enhanced EV secretion with altered EV compositions in various types of liver injury.^[^ ^48^ ^]^ For example, steatotic hepatocytes secrete increased amounts of EVs,^[^ ^14^ ^]^ and cirrhotic hepatocytes release increased amounts of EVs via the autophagic‐lysosomal/multivesicular body and Rab27A pathways.^[^ ^19^ ^]^ In this study, we found higher levels of hepatic EV secretion and upregulated EV biogenesis‐related pathways (e.g., *Rab27a*) in the liver tissues of both patients with cirrhosis and cirrhotic mice. Moreover, hepatic EVs from cirrhotic mice can induce skeletal muscle inflammatory injury in vivo, and this effect can be mitigated by suppressing hepatic EV secretion using specific inhibitors or genetic tools, suggesting that hepatic EV secretion plays a crucial role in mediating skeletal muscle inflammation during cirrhosis.

Endogenous EVs can be actively secreted by various tissue cells and are capable of being internalized by distant recipient cells in vivo. Skeletal muscle tissues contain a diverse array of nonimmune cells (e.g., muscle stem cells and endothelial cells) and immune cells (e.g., monocytes, macrophages, and T cells).^[^ ^5^, ^49^ ^]^ Extensive evidence indicates that macrophages play crucial roles in the pathogenesis of muscle injury and regeneration.^[^ ^49^, ^50^ ^]^ For example, a recent single‐cell RNA‐seq study revealed that the expression of proinflammatory markers (e.g., S100a8 and S100a9) as well as *Fabp4*, *Fabp5*, and *IL‐1b*, was notably upregulated within macrophage clusters in the muscle tissues of aged mice, indicating a pivotal role for macrophages in proinflammatory injury during muscle wasting.^[^ ^50^ ^]^ Currently, research on the alterations in immune cells in cirrhosis‐induced muscle atrophy is limited, but some studies have suggested that persistent inflammation contributes to the onset and progression of chronic obstructive pulmonary disease (COPD) induced sarcopenia. For example, neutrophils can induce muscle damage and impair the response to resistance training in the quadriceps of patients with COPD.^[^ ^51^ ^]^ In our study, the infused hepatic EVs were internalized by skeletal muscle macrophages in vivo and induced a proinflammatory phenotype in macrophages with minimal effects on myotubes. The cGAS–STING pathway plays a key role in regulating the innate immune response and can trigger the release of type I IFNs and many inflammatory factors upon the recognition of exogenous (e.g., bacterial or viral) DNA, thereby exerting antimicrobial effects during infections.^[^ ^52^ ^]^ Abnormal activation of the cGAS–STING pathway has been implicated in various diseases, such as autoimmune diseases, aging‐related inflammation, and neurodegeneration.^[^ ^22^, ^52^ ^]^ Our results also revealed that hepatic EVs could activate the cGAS–STING pathway in skeletal muscle macrophages, while this effect could be partially suppressed by hepatic EV secretion inhibition or STING inhibition, suggesting that hepatic EV secretion induces skeletal muscle inflammatory injury via cGAS‐STING in macrophages. Therefore, STING signaling might be a potential therapeutic target for this disease.

Increasing evidence indicates that EVs can activate the cGAS–STING pathway in diverse cell types during the development of inflammatory diseases and that the mtDNA transferred by EVs is likely one of the primary effectors.^[^ ^53^, ^54^ ^]^ The mitochondrial genome (mtDNA) encodes 13 essential core subunits of respiratory chain complexes, which play crucial roles in regulating cellular metabolism and cell fate.^[^ ^55^ ^]^ However, mitochondrial injury can cause mtDNA instability, oxidative stress, and the innate immune response via the release of cytosolic mtDNA, which is involved in the pathology of many diseases, such as liver cirrhosis.^[^ ^56^ ^]^ On the other hand, mitochondrial stress plays a critical role in regulating EV secretion in diverse cell types.^[^ ^11^, ^57^ ^]^ Additionally, mtDNA‐carrying EVs can impact the metabolic state and immune response of recipient cells in various disease types, such as cancer, aging, and autoimmune diseases.^[^ ^11^, ^29^, ^36^ ^]^ Besides the mtDNA, many other types of EV cargos, such as miRNAs and proteins, might also contribute to muscle injury by inducing metabolic disorders or inflammation. A recent review has summarized the possible pro‐wasting EV cargos that promote the skeletal muscle wasting, such as miRNAs (e.g., miR‐21 and miR‐122) and cytokines (e.g., IL‐6 and Hmgb1).^[^ ^58^ ^]^ For instance, in cancer‐related muscle atrophy, EVs contains Hmgb1 (a proinflammatory protein) derived from colon cancer cells can induce muscle atrophy via activating the TLR4/NF‐κB pathway.^[^ ^59^ ^]^ Similarly, we found that HMGB1 protein levels were also upregulated in the BDL‐EVs compared to the Sham‐EVs (Figure S16, Supporting Information), suggesting that additional bioactive EV cargos (e.g., Hmgb1) carried by hepatic EVs might participant in inducing skeletal muscle inflammation and atrophy development during cirrhosis. Although the effect of hepatic EVs on regulating the liver‐muscle axis during skeletal muscle atrophy development remains to be explored, liver EVs play crucial roles in liver‐to‐organ communication by transferring various types of signaling molecules.^[^ ^48^ ^]^ For example, EVs derived from impaired liver aggravate alveolar bone loss via shuttle of fatty acid synthase (Fasn) which induces the pyroptosis of periodontal ligament cells in type 2 diabetes mellitus.^[^ ^60^ ^]^ However, the analysis of our LC‐MS results did not reveal a significant difference in Fasn expression in liver EVs (Figure S16, Supporting Information), suggesting the specificity of the liver EVs in various liver diseases.

Despite the potential of EV secretion or the STING pathway as therapeutic targets for preventing cirrhosis‐related skeletal muscle atrophy, their clinical translation still requires careful assessment for several reasons. In preclinical studies, a range of methodologies, such as pharmacological interventions (e.g., GW4869) and genetic editing (e.g., Rab27a), have been utilized to reduce EV secretion in vivo.^[^ ^14^, ^18^, ^61^ ^]^ However, these tools are currently limited to animal use and are far from clinical application, with none having been tested for efficacy and biosafety in clinical trials. It is worth exploring the feasibility of human trials. In recent years, gene therapy has emerged as a promising option for treating various liver disorders.^[^ ^62^ ^]^ For example, in clinical trials, the treatment of α1‐antitrypsin deficiency was achieved through the injection of siRNA or antisense oligonucleotides, aimed at reducing the expression of the mutated SERPINA1 gene in hepatocytes and minimizing the accumulation of toxic protein.^[^ ^63^ ^]^ Currently, there are no clinical trials of gene therapy for inhibiting EV secretion. However, suppression of EV secretion using liver‐targeted gene therapy has been shown to exert anti‐inflammatory effects in mouse models,^[^ ^14^, ^61^ ^]^ suggesting the potential for clinical application. Furthermore, previous studies have reported that STING inhibition is an effective strategy for treating various inflammatory diseases in preclinical models, such as rheumatoid arthritis and multiple sclerosis.^[^ ^64^, ^65^ ^]^ A recent clinical study has shown the efficacy of a cGAS‐STING inhibitor (VENT‐03) in attenuating autoimmune diseases, such as systemic lupus erythematosus.^[^ ^66^ ^]^ However, the clinical efficacy of small molecule drugs is often compromised by their suboptimal targeting capabilities in vivo, leading to off‐target effects and reduced therapeutic effectiveness at inflamed sites. To address these issues, researchers have developed targeted drug delivery systems to improve their therapeutic index while reducing side effects in vivo. For example, targeted nanomicelle carriers have been utilized to enhance the bioavailability of a cGAS‐STING inhibitor (RU.521) in inflamed colonic tissue via targeting CD44.^[^ ^67^ ^]^ Besides, other strategies that can interfere with the STING pathway, such as natural products, have been reported. For instance, Ginkgetin has been reported to alleviate inflammation and senescence of macrophages by inhibiting STING,^[^ ^68^ ^]^ which may provide a new avenue for treating muscle atrophy due to its feasibility of clinical translation.

Although these findings are encouraging, several important questions remain unanswered in the current study and need to be addressed in future research. For example, in addition to the use of two mouse cirrhosis models, our results need to be verified using other types of cirrhosis models, large animal models, and samples from cirrhosis patients, as findings from small animal models of cirrhosis are unlikely to be directly translated to human cirrhosis. Whether hepatic EVs also contribute to the pathology of other cirrhosis‐related complications, such as hepatic encephalopathy and cirrhotic cardiomyopathy, remains an open question. In addition to hepatocytes, other cell types within liver tissues, such as immune cells, may also play a proinflammatory role in skeletal muscle tissues, as mitochondrial stress can induce EV secretion in immune cells (e.g., macrophages).^[^ ^69^ ^]^ Although we have demonstrated that mitochondrial injury can promote mtDNA‐enriched EV secretion in hepatocytes, the precise molecular mechanism by which mitochondrial injury regulates the processes of EV biogenesis and cargo sorting under these conditions remains elusive. Previous studies have shown that mitochondrion‐targeted interventions, such as the inhibition of VDAC1 oligomerization and Sirtuin 5‐mediated desuccinylation of ALDH2, can alleviate acute liver injury.^[^ ^70^, ^71^ ^]^ Therefore, it is worth exploring whether these mitochondrial protective tools can be used to reduce hepatic EV‐mediated muscle injury in future studies.

**Scheme 1:**
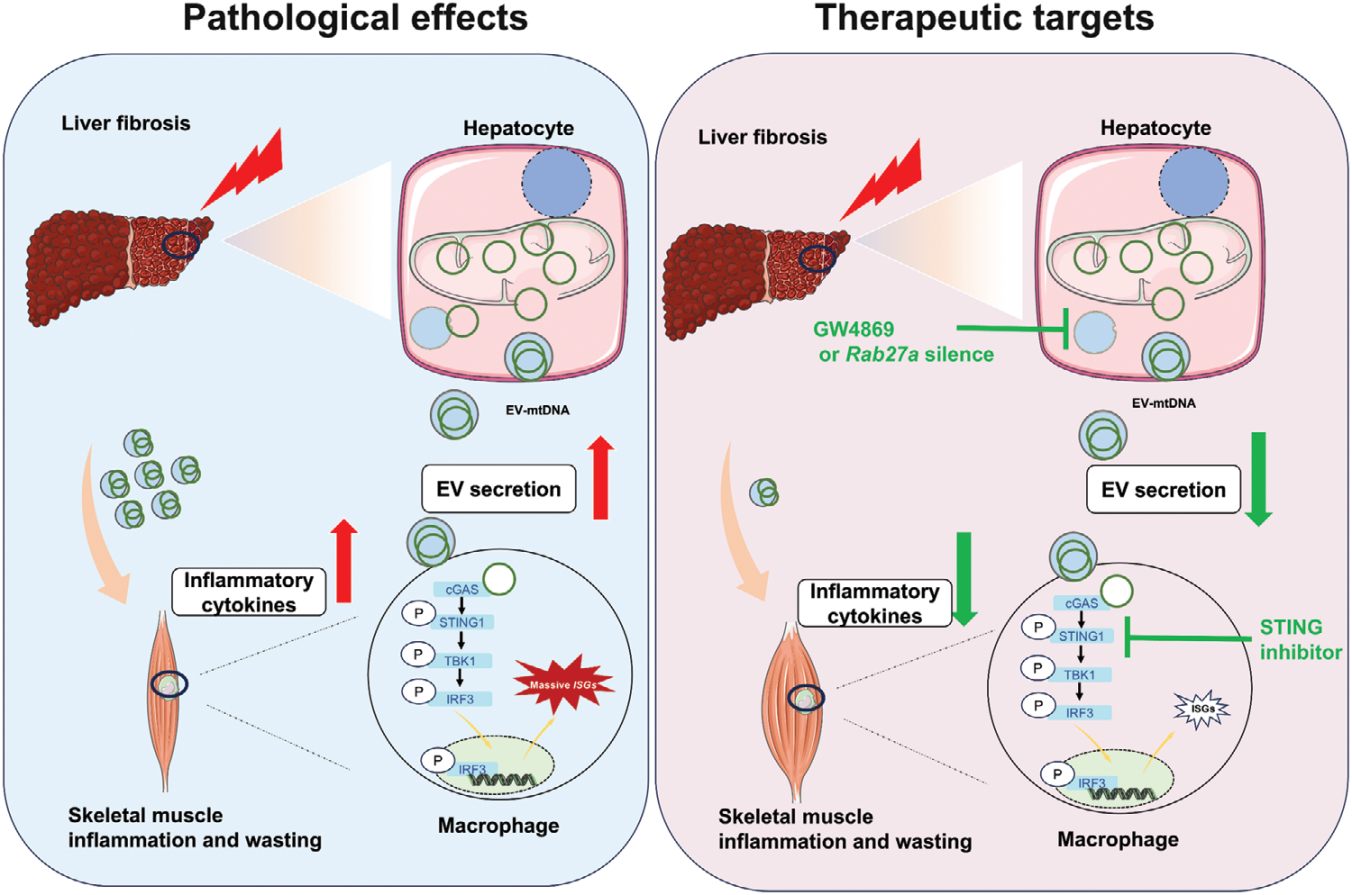
Schematic illustration of injured hepatocytes‐secreted extracellular vesicles promote cirrhosis‐associated skeletal muscle injury through mtDNA‐cGAS/STING axis.

Nevertheless, this study revealed that injured hepatocyte‐derived EVs can promote skeletal muscle inflammation by transferring mtDNA and activating the STING pathway in macrophages, and that targeted inhibition of hepatic EV secretion or STING signaling is a potent therapeutic approach for preventing cirrhosis‐related muscle atrophy (**Scheme** **1**).

## 4 Experimental Section

### Human Studies

The study protocol was approved by West China Hospital's Ethics Committee, and written informed consent was obtained from the patients and volunteers. Consecutive primary biliary cholangitis patients with cirrhosis (*n* = 137) who underwent pretreatment abdominal CT scans (Table S3, Supporting Information) were reviewed through an autoimmune liver disease database from West China Hospital, as previously reported.^[^ ^72^, ^73^ ^]^ The criteria for patient nadir were as follows: 1) Aged 18–75 years; 2) Diagnosis of primary biliary cholangitis patients with cirrhosis; 3) Patients with other liver diseases were excluded; 4) Patients who presented with severe disease or malignant tumors in other systems were excluded. 5) Patients who were pregnant or lactating were excluded. Pretreatment CT images were collected from the electronic medical records system at our institution. Image analysis was conducted by a clinically trained radiologist (L.B.) via Slice‐o‐matic (Tomovision V 5.0). The total skeletal muscle area (SMA) at the third lumbar vertebra (L3) cross‐sectional image was measured, and then the SMA was divided by the height squared (m^2^) to calculate the skeletal muscle index (SMI). Sarcopenia was defined as a SMI < 40.8 cm^2^ m^−2^ for men and <34.9 cm^2^ m^−2^ for women, according to a previous study in the Asian population.^[^ ^74^ ^]^

In patients with cirrhosis who underwent the transjugular intrahepatic portosystemic shunt (TIPS) procedure (*n* = 22), blood was collected from the hepatic veins during the operation (Table S4, Supporting Information). The criteria for patient nadir were as follows: 1) Aged 18–75 years; 2) Patients with liver cirrhosis diagnosed on the basis of clinical manifestations and laboratory and imaging examinations; 3) Patients with previous TIPS or surgical shunts were excluded; 4) Patients with organ dysfunction were excluded; 5) Patients with malignant tumors were excluded. 6) Patients who were pregnant or lactating were excluded. Briefly, the right internal jugular vein was used to let the TIPS set into the inferior vena cava, followed by the right hepatic vein. The portosystemic pressure gradient (PPG) was measured after the portal vein was successfully punctured and catheterized. After the intrahepatic parenchymal tract was predilated with a balloon, the stents were inserted, and the PPG was repeated.

Another cohort of cirrhotic patients (*n* = 18) was recruited on the basis of histological, biochemical, and imaging evidence at West China Hospital. The criteria for patient nadir were as follows: 1) Aged 18–75 years; 2) Patients with liver cirrhosis diagnosed on the basis of clinical manifestations and laboratory and imaging examinations; 3) Patients with malignant tumors were excluded; 4) Patients who were pregnant or lactating were excluded. Healthy controls (*n* = 13) were recruited during the medical examination. Elbow vein blood was obtained, and serum EVs were collected. Two experienced operators performed 2D shear wave elastography (2D‐SWE) on a supersonic imaging Aixplore ultrasound system. 2D‐SWE measurements were performed from the right hepatic lobe to the intercostal spaces. For graphing, SWEs were collectively referred to as LSMs.

### Isolation of Serum EVs

Serum was collected from human blood by serial centrifugation at 1500 × g for 10 min and 3000 × g for 15 min at 4 °C. Then, EVs were extracted from the serum via the exoEasy Maxi Kit (76064, QIAGEN, USA).

### Animal Models and Treatment

Male C57BL/6 mice (aged 6–8 weeks) were purchased from GemPharmatech Co., Ltd. (Nanjing, China) and were maintained in a specific pathogen‐free facility. All experiments were carried out according to protocols approved by the Animal Ethics Committee of West China Hospital, Sichuan University. (No. 20230308010). At the indicated time points, the mice were sacrificed, and their livers, muscles, and sera were collected for further assays. Serum AST, CK, and LDH levels were detected using an automatic biochemical analyzer (Cobas8000 702, Roche, Switzerland).

For the CCl_4_‐induced liver fibrosis model (oil group and CCl_4_ group, *n* = 5), the mice were intraperitoneally (i.p.) injected with CCl_4_ (1.0 mL kg^−1^ body weight, dissolved in Olivia oil at a ratio of 1:4) or vehicle (Olivia oil) twice a week for 12 weeks. For the BDL‐induced liver fibrosis model (sham group and BDL group, *n* = 5), the mice were subjected to either BDL or a sham operation for 14 days.

To test the in vivo effect of hepatic EVs, after 1 week of BDL or sham operation, WT mice were treated with an intramuscular injection of Sham‐EVs or BDL‐EVs (1 × 10^10^ particles mL^−1^) three times a week. One week after EV transfer, the mice were starved overnight and sacrificed for analysis (Sham‐EVs group and BDL‐EVs group, *n* = 6).

For the inhibition of hepatic EV release and STING signaling in vivo, different treatments were performed. For GW4869 treatments, BDL or sham mice were intraperitoneally (i.p.) injected with GW4869 (1.25 mg kg^−1^, CSN Pharma, USA) or vehicle (dimethyl sulfoxide) for 1‐week postsurgery (Vehicle group and GW4869 group, *n* = 5). For liver‐specific *Rab27a* interference, the targeting sequence for *Rab27a* knockdown was GCTTCTGTTCGACCTGACAAA. The mice were injected with AdV‐sh*Con* or AdV‐sh*Rab27a* (1 × 10^9^ PFU per mouse, Genechem, Shanghai, China) via the tail vein, and then, BDL surgery was performed (group sh*Con* and group sh*Rab27a*, *n* = 6). Mice were starved overnight and sacrificed for analysis after 14 days. For STING inhibition, mice were subjected to intramuscular injection of H‐151 (210 µg per mouse, TargetMol, USA) for 1 week postsurgery (group Vehicle and group H‐151, *n* = 5). For NF‐κB inhibition, the mice were subjected to intramuscular injection of Bay 11‐7085 (25 mg kg^−1^, TargetMol, USA) for 1 week after surgery (vehicle and Bay 11‐7085 groups, *n* = 6). For chemotaxis inhibition, the mice were subjected to intramuscular injection of a CCR2/CCR5 inhibitor (cenicriviroc, 50 mg kg^−1^; TargetMol, USA) for 1 week postsurgery (vehicle group and cenicriviroc group, *n* = 6).

### Grip Strength Test

The mice were placed on a wire grid. The amount of force (Newton, N) applied to the grid each time before the mouse lost its grip on the grid was recorded. Three repeats were performed for each mouse, and the averaged data were calculated and used.

### Isolation of Tissue‐Derived EVs

After the mice were anesthetized, they were injected via the portal vein for liver perfusion with HBSS basic (Gibco, USA). Then, collagenase IV (1 mg mL^−1^ in Hanks balanced salt solution (Beyotime, China)) was used to digest the liver tissue. After collagenase digestion, a large volume of HBSS was added for neutralization. This was followed by serial centrifugation at 50 × g for 10 min to remove tissue masses, 300 × g for 10 min and 2000 × g for 20 min at 4 °C to remove other cells. The supernatant was subsequently ultracentrifuged at 10 000 × g for 70 min at 4 °C to remove cell debris. Next, the supernatant was ultracentrifuged at 100 000 × g for 70 min at 4 °C, and the isolated EVs pellets were washed by resuspension in PBS and then subjected to a second round of ultracentrifugation. Finally, the pure EVs pellets were resuspended in PBS and stored at −80 °C for subsequent experiments. Tissue‐derived EVs from the mouse heart, lung, and kidney were isolated as previously reported.^[^ ^75^ ^]^

### Transmission Electron Microscopy

The morphology of the EVs and livers was evaluated via TEM (JEM‐1400PLUS, JEOL, Japan). EVs samples were diluted with PBS and dropped on a copper grid. After dehydration, embedding, and ultrathin slicing, the grid was stained with 2% phosphotungstic acid and then subjected to TEM.

### Nanoparticle Tracking Analysis

The size and amount of EVs were detected by NTA (Zeta View PMX 120, Particle Metrix, Germany). EVs were diluted 3000 times with pure water. The parameters were as follows: sensitivity: 80, shutter: 100, minimum brightness: 20, minimum area: 5, and maximum area: 1000 at 25 °C.

### Biodistribution of Hepatic EVs in Mice

EVs were labeled with sulfo‐Cy7‐NHS ester (Little‐PA Sciences, Wuhan, China). Male C57BL/6 mice were intraperitoneally injected with Cy7‐EVs (100 µg protein per mouse), and mice receiving equal amounts of free Cy7 solution were used as dye controls. After 1 h, the mice were sacrificed, and the muscles (diaphragm, abdominal, quadriceps, tibialis anterior, and gastrocnemius) were collected and observed via an optical imaging system (IVIS Spectrum, USA**)**.

### Liver/Muscle Histology and Immunostaining

The tissues were fixed in 4% paraformaldehyde, embedded in paraffin, and sliced into 4 µm thick sections. Then, the liver sections were subjected to H&E, Masson's trichrome, and Sirius Red staining, and the muscle sections were subjected to H&E staining. For immunohistochemistry, the muscle sections were incubated with primary antibodies against CD3 (GB12014, Servicebio), CD4 (GB15014, Servicebio), CD8 (GB15068, Servicebio), CD45 (GB113886, Servicebio), and CD11B (GB11058, Servicebio) at 4 °C overnight; the muscle sections were incubated with primary antibodies against RAB27A (EM1706‐32, HuaBio) at 4 °C overnight. After being washed with PBS, the sections were stained with HRP‐conjugated secondary antibodies and DAB. Images of the stained sections were quantified via ImageJ software.

### Cell Culture and Treatment

C2C12 myoblasts, RAW264.7 cells, and AML12 cells were cultured in Dulbecco's modified Eagle's medium (DMEM, Gibco, CA, USA) supplemented with 10% FBS (Corning, NY, USA) and 1% penicillin‐streptomycin (MedChemExpress, NJ, USA). The differentiation of C2C12 myoblasts was induced by changing the 10% FBS mixture to 2% horse serum for 3–5 days. All cells were cultured at 37 °C with 5% CO_2_. C2C12‐derived myotubes were treated with macrophage‐conditioned medium preincubated with 0.1% Triton X‐100 or 0.1% Triton X‐100 and DNase. C2C12‐derived myotubes or macrophages were treated with Sham‐EVs or BDL‐EVs (6 × 10 × ^10^ mL^−1^) for 24 h. The macrophage‐conditioned medium was centrifuged at 3000 × g for 40 min with centrifugal filter unit (15 mL, 3 kDa MWCO, Millipore), and concentrated three times.

### Deletion of mtDNA Contents in EVs

The isolated EVs were preincubated with 0.1% Triton X‐100 for 0.5 h, and then treated with DNase at 37 °C for 0.5 h as previously reported,^[^ ^29^ ^]^ according to the manufacturer's instructions.

### Isolation of Cell‐Derived EVs

EV‐depleted FBS was prepared by ultracentrifugation (120 000 × g, 16 h, 4 °C). Then, the AML12 cells were cultured in DMEM supplemented with 10% EV‐depleted FBS. EVs were isolated from cell conditioned medium via a differential centrifugation method. In brief, the medium was centrifuged at 300 × g for 10 min, 2000 × g for 20 min, and 10 000 × g for 30 min at 4 °C to remove cells and other debris, followed by ultracentrifugation at 100 000 × g for 90 min at 4 °C in an SW32Ti rotor (Beckman Coulter, USA) to obtain EVs. After washing with PBS, the EV pellets were recentrifuged at 120 000 × g for 90 min at 4 °C. The purified EVs were resuspended in PBS and stored at −80 °C until further use.

### Uptake of EVs in Cells

EVs were labeled with PKH26 (UR52302, Umibio) according to the manufacturer's protocol. The stained EVs were purified and resuspended in PBS for use in cell uptake experiments. RAW264.7 cells or C2C12‐derived myotubes were incubated with PKH26‐labeled EVs at 37 °C. After being washed with PBS, the cells were fixed with 4% paraformaldehyde for 15 min and then stained with FITC‐phalloidin at room temperature. Nuclei were visualized by staining with DAPI. Images of the stained cells were obtained using a confocal laser scanning microscope (N‐STORM).

### Cell Viability Assay

The cells were seeded into 96‐well plates and cultured overnight. The next day, 10 µL of 5 mg mL^−1^ MTT (Sigma‒Aldrich, USA) was added to each well. After incubation for 4 h at 37 °C, 150 µL of DMSO was added to each well. The plates were subsequently shaken at low speed for 10 min. After the purple crystallites were fully dissolved, the absorbance was recorded at 490 nm.

### Flow Cytometry Analysis

After the cells were collected and suspended in PBS, they were incubated with anti‐mouse MHC II and anti‐CD86 antibodies (BioLegend, USA) at 4 °C for 30 min. C2C12 cell apoptosis was assessed via the Annexin V‐FITC/PI staining method with an Annexin V‐FITC/PI cell apoptosis detection kit (Servicebio, G1511‐100T, China) according to the manufacturer's guidelines. Apoptosis was quantified via a flow cytometer (Beckman, USA).

### Mitochondrial and Cytoplasmic dsDNA Assay

For colocalization of dsDNA and mitochondria in AML12 cells, MitoTracker Red (200 nm, Yeason, China) and PicoGreen (500 ng mL^−1^, Yeason, China) were added to DMEM. After that, images of the stained cells were obtained via a confocal laser scanning microscope (Nikon N‐STORM, USA).

### Detection of the Mitochondrial Membrane Potential

The mitochondrial membrane potential of the AML12 cells was assessed via a JC‐1 Assay Kit (Beyotime Biotechnology, China). In brief, AML12 cells were treated with APAP (MedChemExpress, USA) and subjected to a 20 min incubation with JC‐1 dye at 37 °C. Following a wash step with JC‐1 staining buffer, the JC‐1 fluorescence of the AML12 cells was observed via a fluorescence microscope (Zeiss, Germany).

### Immunofluorescence Staining

The cells or frozen sections of muscle tissues were fixed, permeabilized, blocked and then incubated overnight at 4 °C with diluted primary antibodies against F4/80 (GB113373, Servicebio), P‐STING (YP1518, Immunoway), and iNOS (ER1706‐89, HuaBio), followed by incubation with a FITC‐conjugated secondary antibody (Abcam). After washing with PBS and staining with DAPI, images from immunofluorescence staining were analyzed via a confocal laser scanning microscope (N‐STORM) and processed via ImageJ.

### Western Blotting

Proteins were extracted from cells, tissues, or EVs in RIPA lysis buffer (Beyotime, China) supplemented with protease and phosphatase inhibitors (Selleckchem, USA). The protein concentration was calculated via a BCA protein assay kit (Beyotime, China). The extracted proteins were separated by 10% SDS‒PAGE and transferred to PVDF membranes (Millipore, USA). After blocking with 5% milk for 1 h, the membranes were incubated with primary antibodies (anti‐TSG101 (ET1701‐59, HuaBio), anti‐calnexin (ET1611‐86, HuaBio), anti‐ALIX (ET1705‐74, HuaBio), anti‐HSP70 (ET1601‐11, HuaBio), anti‐MuRF1 (ab183094, Abcam), anti‐α‐tubulin (11224–1‐AP, Proteintech), anti‐GAPDH (ET1601‐4, HuaBio), anti‐alpha smooth muscle actin (ET1607‐53, HuaBio), anti‐STING (300415, Zenbio), anti‐phospho‐STING (YP1518, Immunoway), anti‐TBK1 (HA601045, HuaBio), anti‐phospho‐TBK1 (R30260, Zenbio), anti‐IRF3 (ET1612‐14, HuaBio), and anti‐phospho‐IRF3 (ET1608‐22, HuaBio)) at 4 °C overnight. After that, the membranes were incubated with secondary antibodies (Proteintech, SA00001‐2) at room temperature for 1 h. The immunoreactive proteins were detected with an ultrahigh‐sensitivity enhanced chemiluminescence (ECL) kit (MedChemExpress, USA). The expression level was quantified using ImageJ software.

### Quantitative Reverse Transcriptase PCR (qRT‒PCR)

RNA from tissue or cells was isolated with an RNA extraction kit (Axygen, USA), followed by cDNA synthesis via the PrimeScript RT reagent kit (Takara, Japan). Real‐time PCR was carried out by using a Bio‐Rad CFX96 instrument (Bio‐Rad, Hercules, CA, USA). The GAPDH or 𝛽‐actin RNA expression level was used to normalize the data. The primer sequences are listed in Tables S1 and S2 (Supporting Information).

### DNA Isolation and mtDNA Copy Number Assay

Relative levels of mitochondrial DNA (mtDNA copy number) were determined by qPCR. Briefly, total DNA was extracted from EVs via a Universal Genomic DNA Kit (CW2298S, CWBIO, China), and 10 ng of DNA was used for qPCR analysis. For human EVs, the mitochondrial CO_2_ gene (mtCO_2_) was used to measure the mtDNA copy number, which was normalized to that of nuclear beta‐2 microglobulin (B2M). For mouse EVs, the mtDNA copy number was normalized to that of the nuclear ribosomal protein s18 (rps18). The sequences of the primers used in the mtDNA assay are shown in Table S5 (Supporting Information).

### RNA‐Sequence Analysis

After the muscles were injected with Sham‐EVs or BDL‐EVs, total RNA was extracted from the tissue samples via TRIzol. RNA‐seq (Sham‐EVs group and BDL‐EVs group, *n* = 6) was conducted by Biomarker Technology Corporation (Beijing, China). Differentially expressed genes were identified via the DESeq2 package, and functional enrichment for GO and KEGG analyses was performed with the GO stats package in R 4.3.1 (Version 4.2.0; R Foundation, Vienna, Austria).

### Proteomic Analysis of Tissue EVs

Proteomic analysis of tissue EVs (Sham‐EVs group and BDL‐EVs group, *n* = 5) was conducted by APExBIO Technology LLC (Shanghai China). In brief, total proteins were extracted from EVs and refined into peptides. Label‐free proteomic analysis of tissue EV samples was performed via liquid chromatography‐tandem mass spectrometry (LC–MS/MS) detection system (Thermo Fisher Scientific). The raw data were subsequently searched in the database. DEGs (FC > 1.5 and *p*‐value < 0.05) were considered significant. Principal component analysis (PCA) plots, volcano plots, and heatmaps were generated via a free online platform (https://cloud.apexbio.cn/).

### Gene Expression Omnibus Database Analysis

Datasets from the Gene Expression Omnibus (GEO) database was analyzed with GEO2R to profile gene expression between samples, such as normal versus cirrhosis patients (GSE25097). GO enrichment analysis and heatmap construction were performed via R 4.3.1. R (Version 4.2.0; R Foundation, Vienna, Austria).

### Statistical Analysis

All the data are presented as mean ± SEM. The normality of the distribution was assessed via the Shapiro‒Wilk test. Differences between the two groups were assessed via unpaired two‐tailed Student's *t*‐test. Differences between multiple groups were assessed via one‐way ANOVA with Tukey's post hoc test or two‐way ANOVA with Tukey's multiple comparisons test. K‒M survival curves were used to compare the prognosis between the two groups of cirrhotic patients with or without sarcopenia via IBM SPSS Statistics 27. Pearson correlation was used to assess the linear correlation between groups. A *p*‐value < 0.05 was considered to indicate statistical significance. *p < 0.05, **p < 0.01, ***p < 0.001, ****p < 0.0001. GraphPad Prism 9.0.0 software was used for statistical analysis.

## Conflict of Interest

The authors declare no conflict of interest.

## Author Contributions

X.F., Y.P., and B.L. contributed equally to this work. Project conception and experiment design. L.Y. and J.L. Experimental operation. X.F., Y.P., B.L., Y.L., X.W., L.Z., Q.D., and Y.Z. Sample collection. Y.S., G.L., and X.W. Data analysis. X.F., Y.P., and B.L. Writing—original draft. X.F and Y.P. Writing—review and editing. L.Y. and J.L. All experiments were conducted under the supervision of J.L. All authors contributed to design development, data interpretation, and manuscript editing work. Some experimental schemes were created with BioRender.com (https://biorender.com/), and all were confirmed of publication and licensing rights. The authors also thank Servier Medical Art (http://smart.servier.com) for providing elements of the illustrations.

## Supporting information



Supporting Information

Supplemental Table 1

Supplemental Table 2

## Data Availability

All data needed to evaluate the conclusions in the paper are present in the paper and/or the suppplementary Materials.
