# Supplementary material for: Liver‐Secreted Extracellular Vesicles Promote Cirrhosis‐Associated Skeletal Muscle Injury Through mtDNA‐cGAS/STING Axis
Source: Adv Sci (Weinh). 2025 Jan 13;12(9):2410439. doi: 10.1002/advs.202410439 (PMC11884600; doi:10.1002/advs.202410439)
Supplement: Supplementary file 1 — Supporting Information [file ADVS-12-2410439-s001.docx]

**Supplementary** **Materials** **for**

**Liver-secreted extracellular vesicles promote cirrhosis-associated skeletal muscle injury through mtDNA-cGAS/STING axis**

Xiaoli Fan *et al.*

Corresponding author:

Li Yang, Professor, M.D. Email: yangli_hx@scu.edu.cn

Jingping Liu, Professor, Ph.D Email: liujingping@scu.edu.cn

**The PDF file includes:**

Supplementary Methods

Figs. S1 to S16

Tables S1 to S5

Figure legend for Table S6 to S7

**Other Supplementary Material for this manuscript includes the following:**

Tables S6 to S7

**Supplementary methods**

**Cirrhosis model**

For the CCl_4_-induced mouse liver fibrosis model, mice were intraperitoneally (i.p) injected with carbon tetrachloride (CCL_4_, 1.0 mL/kg body weight, dissolved in olivia oil at a ratio of 1:4 (volume/volume), Adamas, China) twice a week for 12 weeks, while mice of Sham group were i.p injected with the same volume of olive oil. For the BDL-induced liver fibrosis model, mice were anaesthetized using isoflurane. Then, the common bile duct was separated from the portal vein and ligatured with sterile silk sutures, while the bile ducts of the Sham group were exposed after laparotomy without ligation. The mice were subjected to either BDL or a Sham operation for 14 days.

**Supplementary Figures**

**
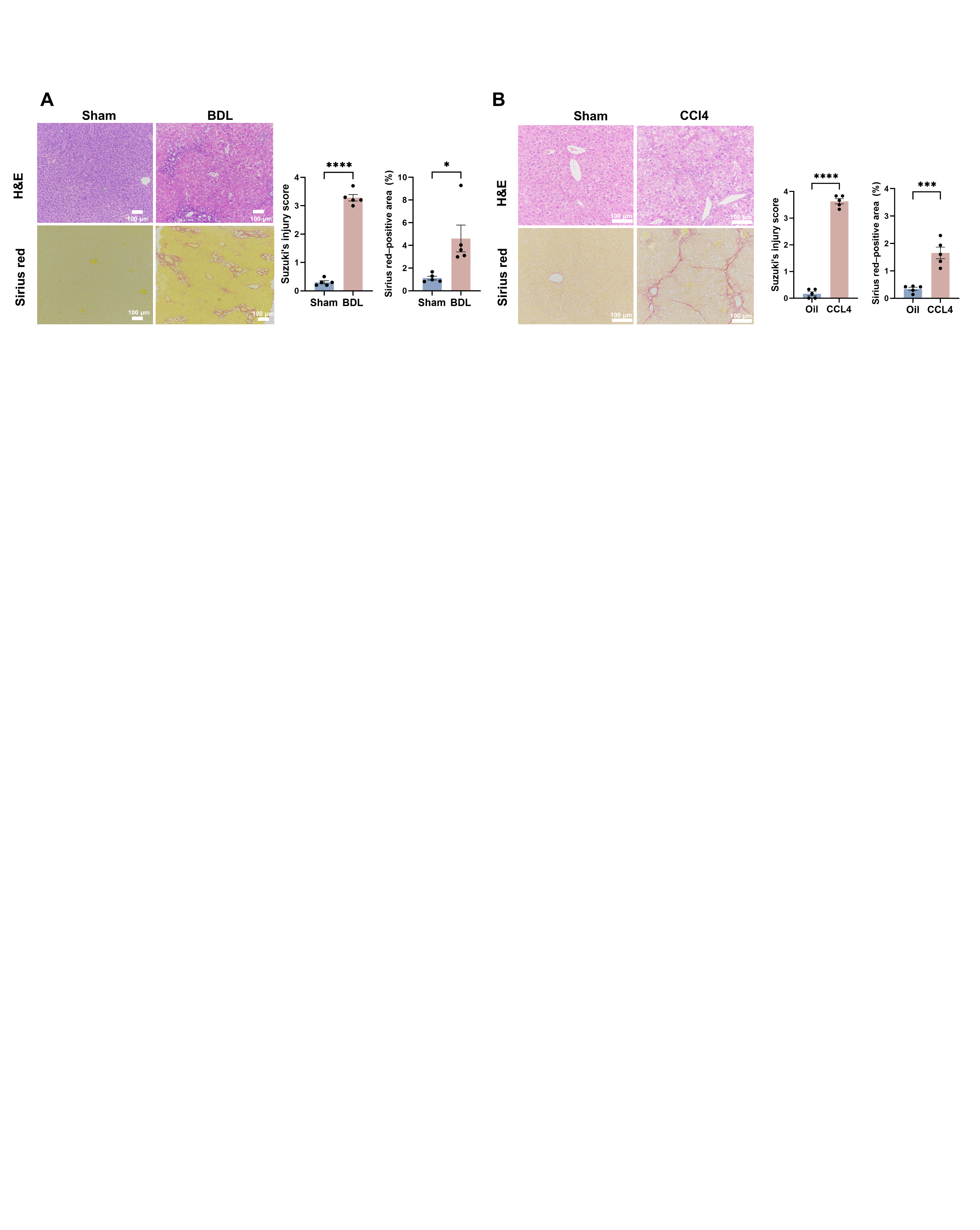
**

**Figure S1 Liver inflammation and fibrosis of BDL and CCL_4_ mice.** (A) Representative liver histology of H&E, Sirius Red staining and the quantification analysis in Sham and BDL mouse livers (scale bar = 100 μm, n = 5). (B) Representative liver histology of H&E, Sirius Red staining and the quantification analysis in Oil and CCL_4_ mouse livers (scale bar = 100 μm, n = 5). *p < 0.05, ***p < 0.001, ****p < 0.0001.


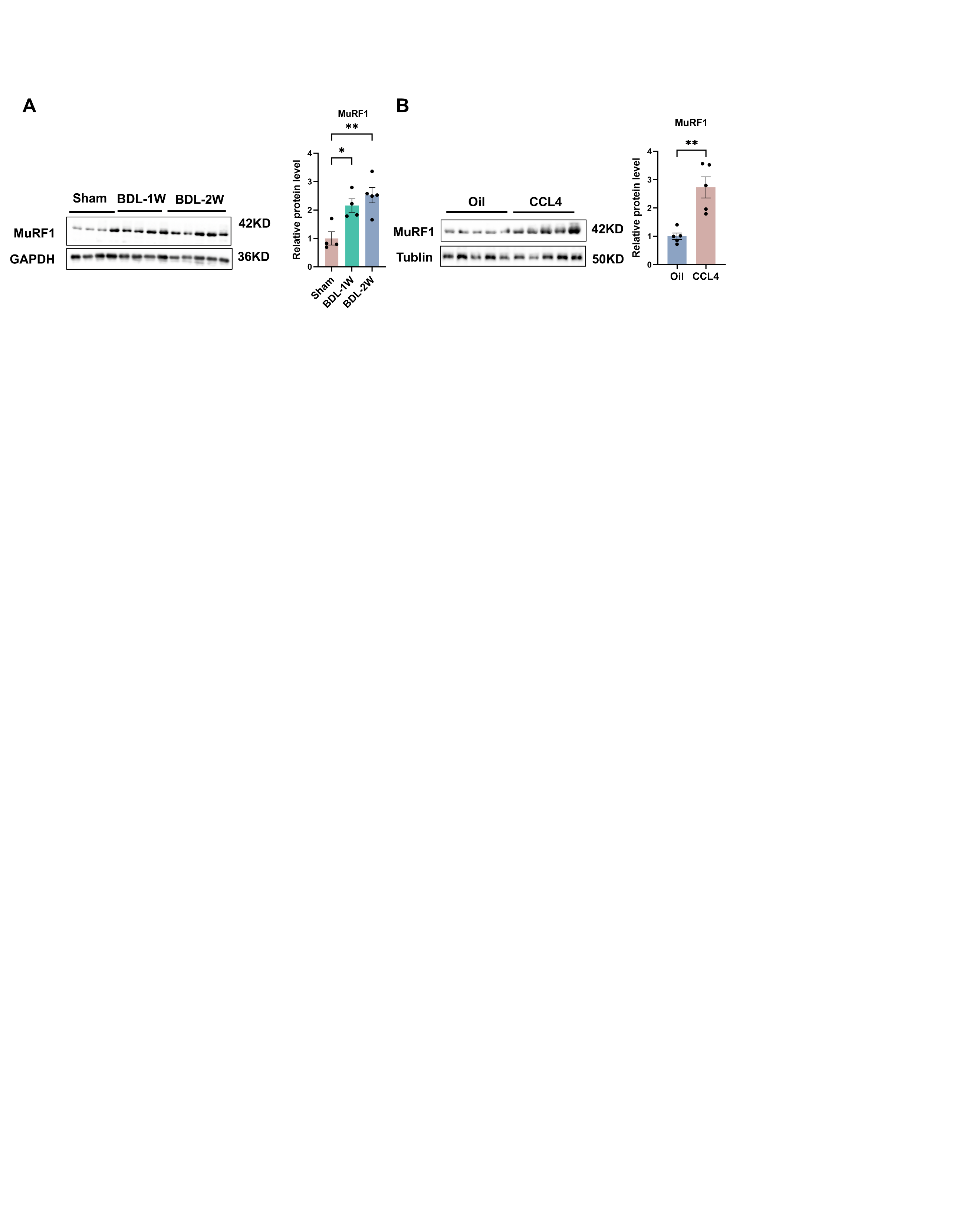


**Figure S2** Representative blots and quantified results of the expression of MuRF1 protein in muscle tissues in BDL or CCL_4_ models (n = 4-5). *p < 0.05, **p < 0.01.


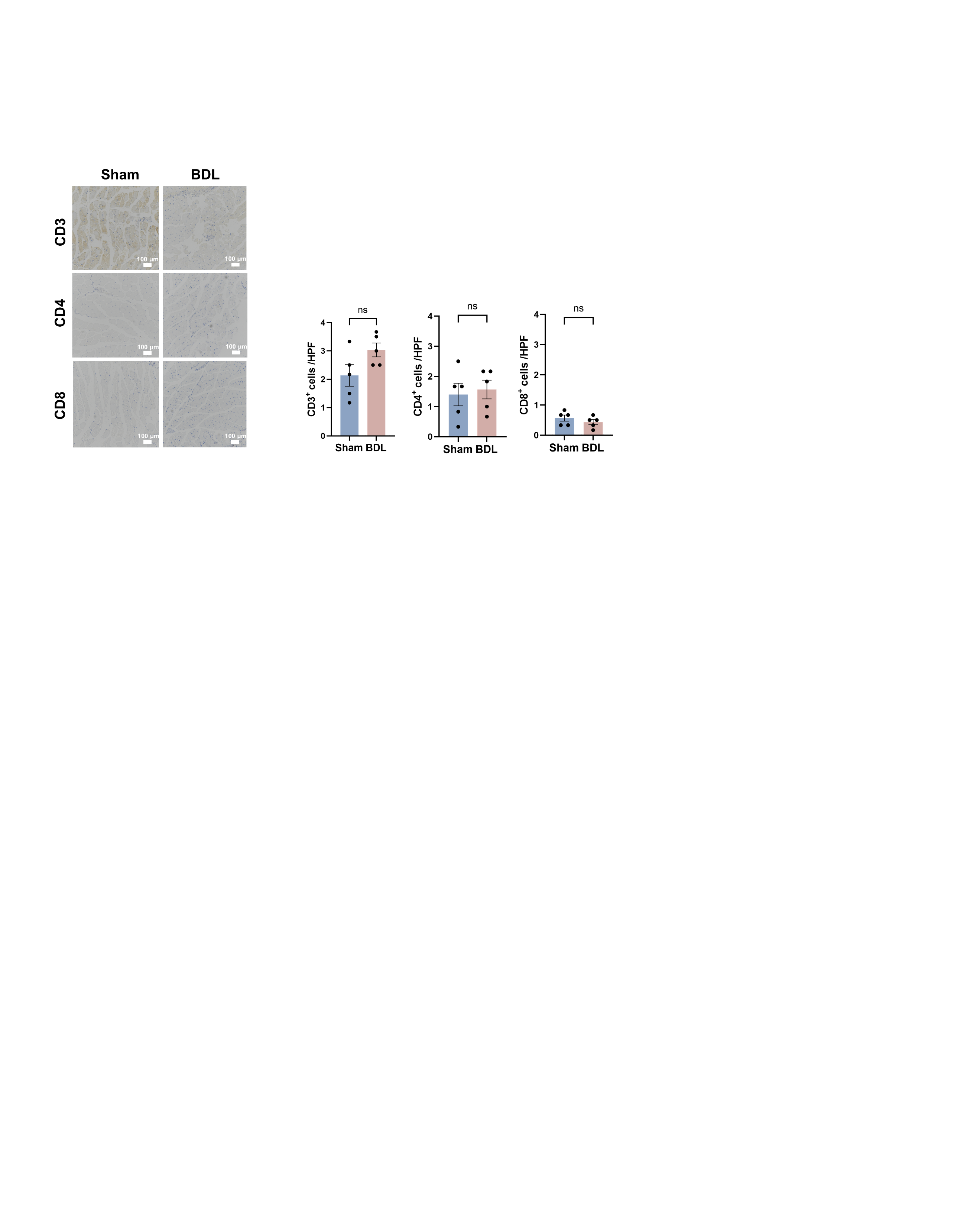


**Figure S3** Representative images of IHC staining (CD3, CD4, CD8) and the numbers of CD3-positive cells, CD4-positive cells, and CD8-positive cells in muscle tissues of BDL model (scale bar = 100 μm, n = 5). ^ns^p ≥ 0.05.

**
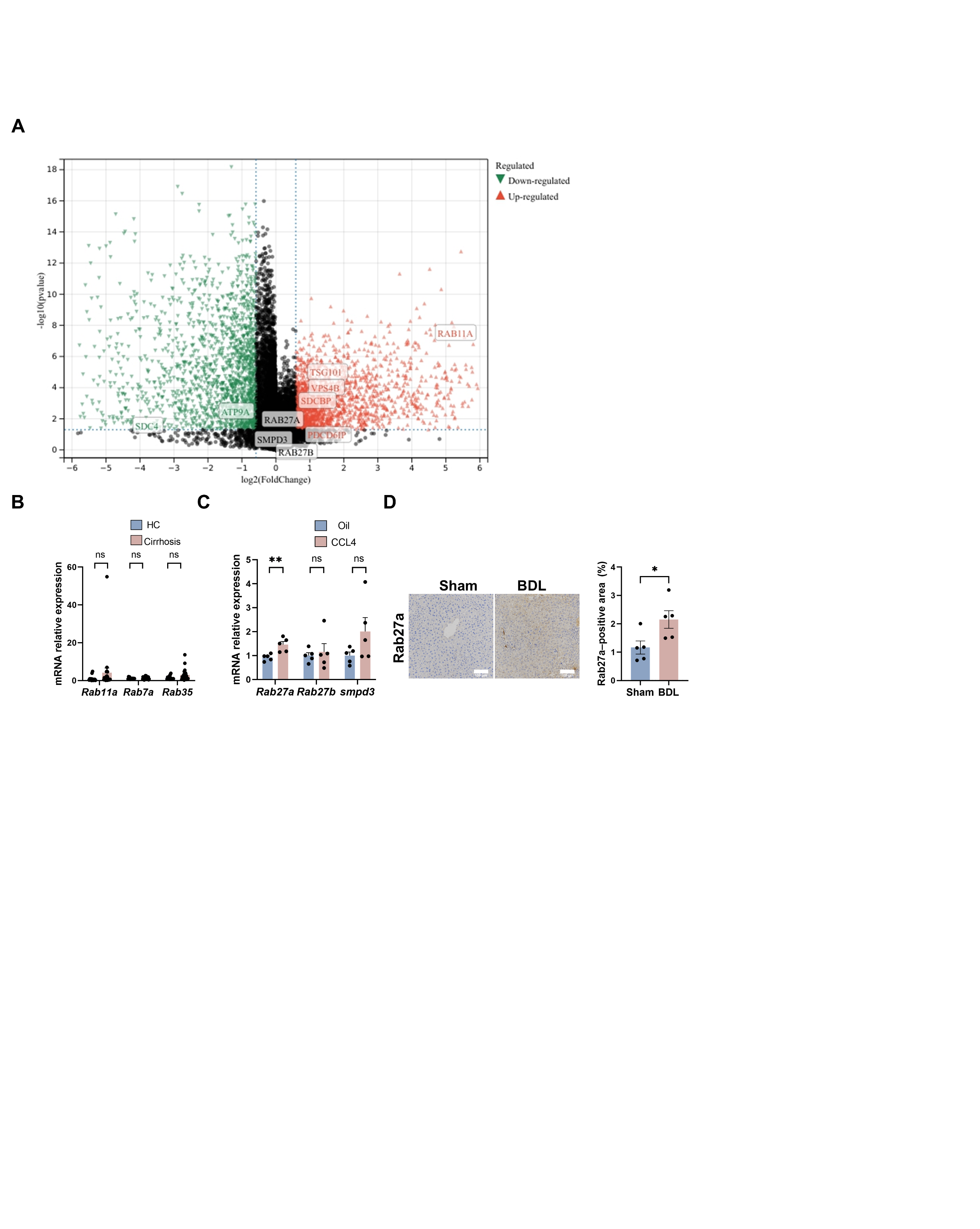
**

**Figure S****4 Analysis of the GSE25097 dataset and validation.** (A) Volcano maps showing the different gene expression profiles based on the GSE25097 dataset. (B) Expression levels of Rab11a, Rab7a and Rab35 in the human liver measured by qPCR (n = 14/22). (C) Expression levels of Rab27a, Rab27b, and smpd3 in the livers of Oil and CCL_4_ mice were measured via qPCR (n = 5). (D) Representative images of IHC staining (Rab27a) and quantification of the positive area in the sham and BDL mice (scale bar = 100 μm, n=5). ^ns^p ≥ 0.05, *p < 0.05, **p < 0.01


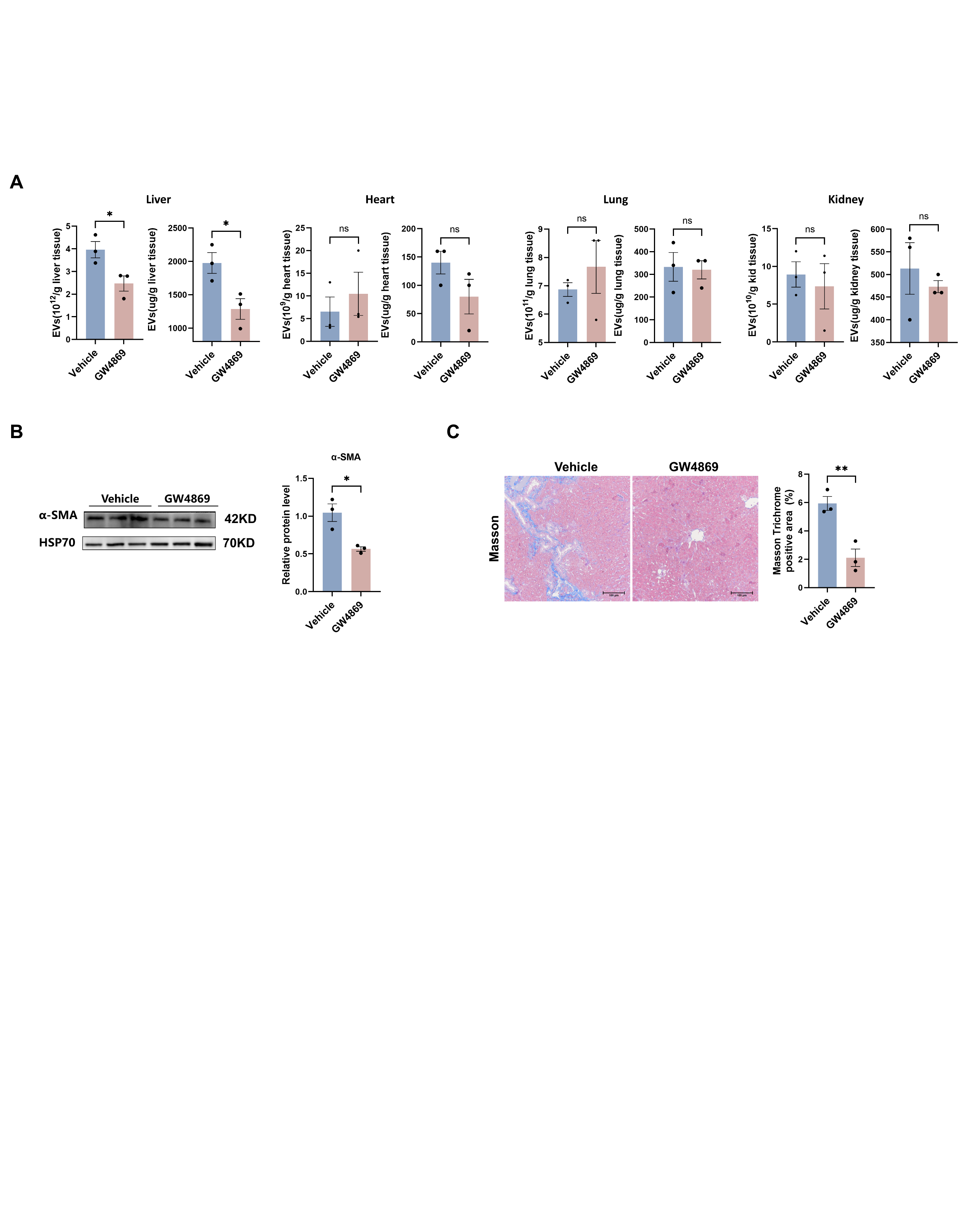


**Figure S5 Effect of GW4869 on EV generation and hepatic fibrosis of BDL mice.**

(A) Measurements of organ EV secretion in the liver, heart, lung and kidney with or without systemic GW4869 treatment (n = 3) (B) Representative blots and quantiﬁed data of the expression of α-SMA protein (n = 3). (C) Representative liver histology of Masson Trichrome staining and its quantification analysis (scale bar = 100 μm, n = 3). *p < 0.05, **p < 0.01.

**
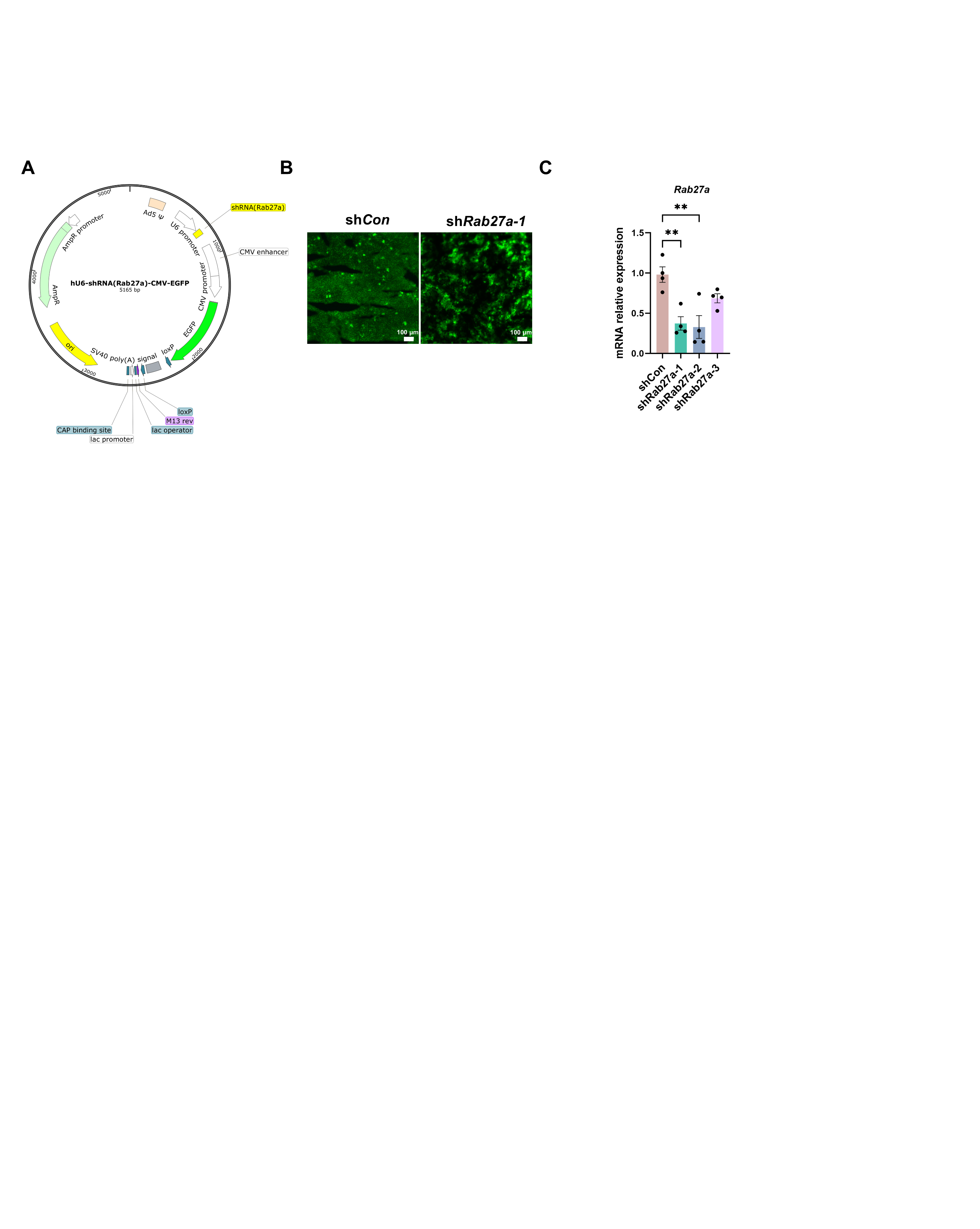
**

**Figure S6 Construction and validation of adenoviral vector-mediated *Rab27a* knockdown.** (A) The construction process of AdV-sh*Rab27a*. (B) Representative images of frozen mouse liver sections with treatments of adenovirus via tail vein (scale bar = 100 μm). (C) Expression levels of *Rab27a* measured by qPCR (n = 4). **p < 0.01.

**
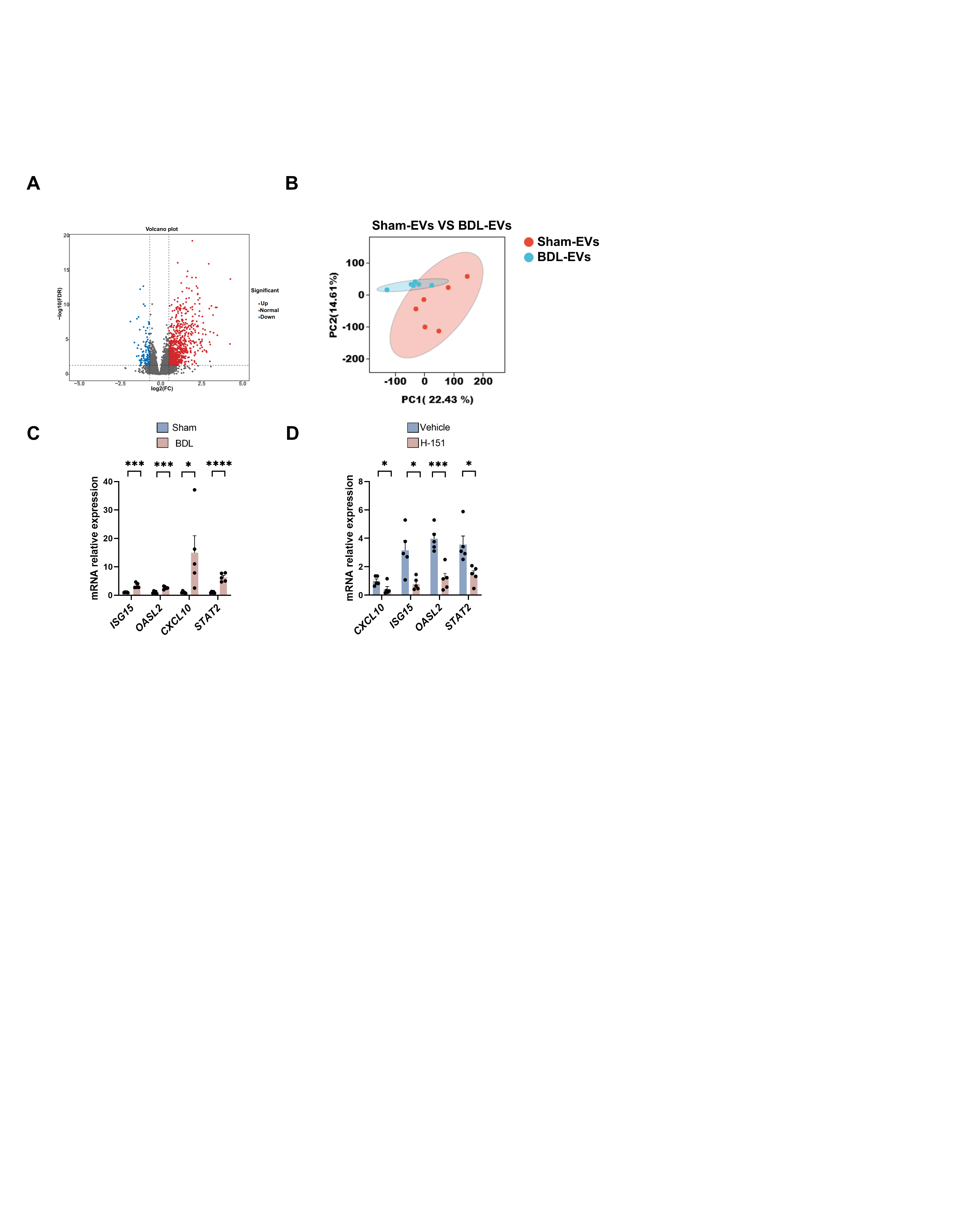
**

**Figure S7 Effect of BDL-EVs on inducing the expression of ISGs in muscles.**

(A) volcano plots showing the DEGs (FC > 1.5 and P- adjusted < 0.05) between the Sham-EVs group and the BDL-EVs group on muscles (n = 6). (B) PCA scatterplot of different groups based on proteomic data showing the differences between different groups (n = 6). (C) Expression levels of *ISG15, OASL2, CXCL10, STAT2* measured by qPCR in Sham and BDL mice (n = 5). (D) Expression levels of *ISG15*, *OASL2*, *CXCL10*, *STAT2* measured by qPCR. (n = 5). ^ns^p ≥ 0.05, *p < 0.05, **p < 0.01, ***p < 0.001, ****p < 0.0001.


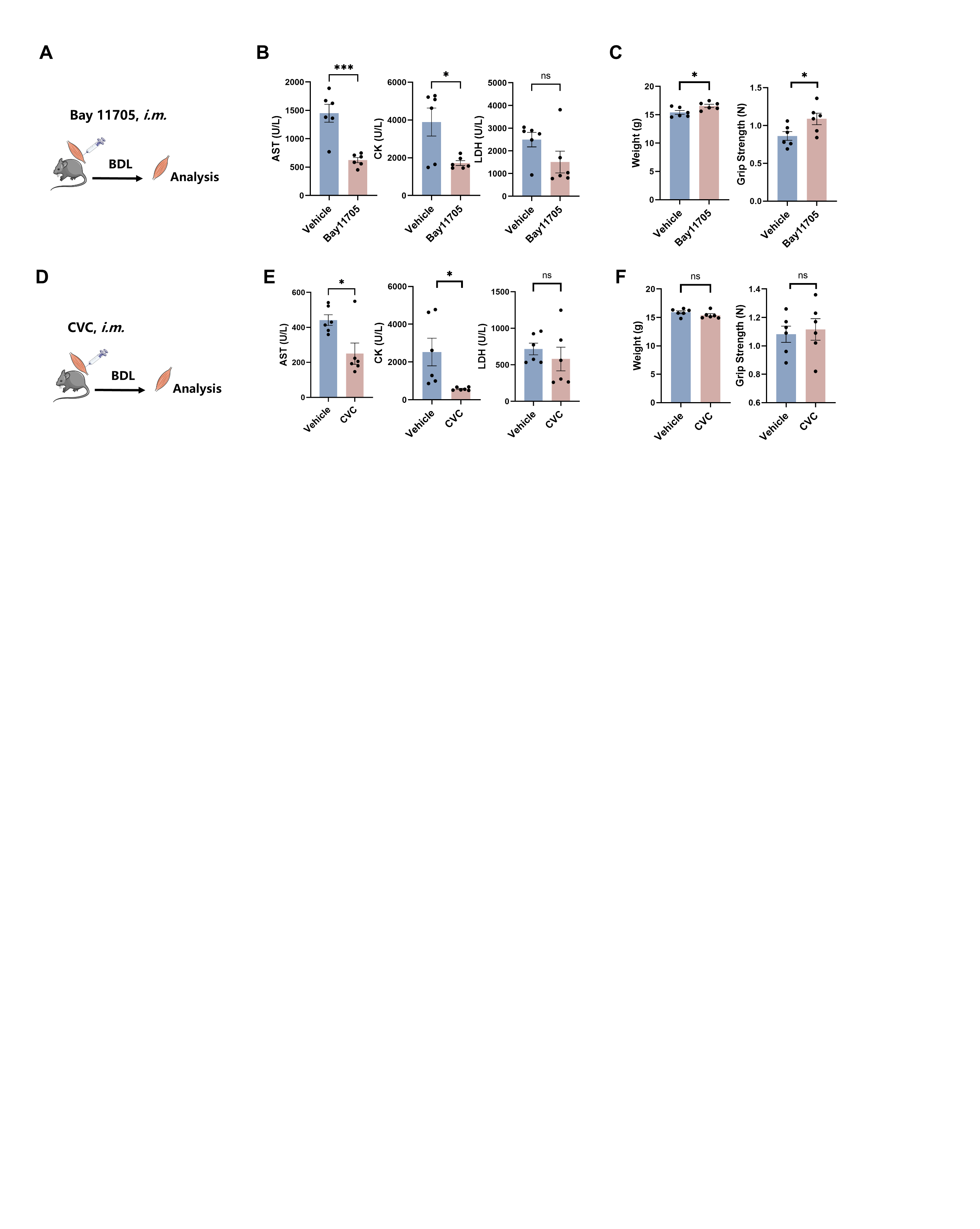


**Figure S8 Effects of NF-κB and chemotaxis pathway inhibition on the skeletal muscle atrophy and inflammation in BDL mice.** (A) Experimental scheme. After the BDL operation, the mice were intramuscularly injected with Bay11705 or vehicle. (B) Serum levels of AST, CK and LDH in vehicle-treated or Bay11705-treated mice (n = 6). (C) Body weight and grip strength of vehicle-treated or Bay11705-treated mice (n = 6). (D) Experimental scheme. After the BDL operation, the mice were intramuscularly injected with CVC or vehicle. (E) Serum levels of AST, CK and LDH in vehicle-treated or CVC-treated mice (n = 6). (F) Body weight and grip strength of vehicle-treated or CVC-treated mice (n = 6). The data are presented as the mean ± s.e.m. ^ns^p ≥ 0.05, *p < 0.05, ***p < 0.001; two-tailed unpaired t test for comparison.

**
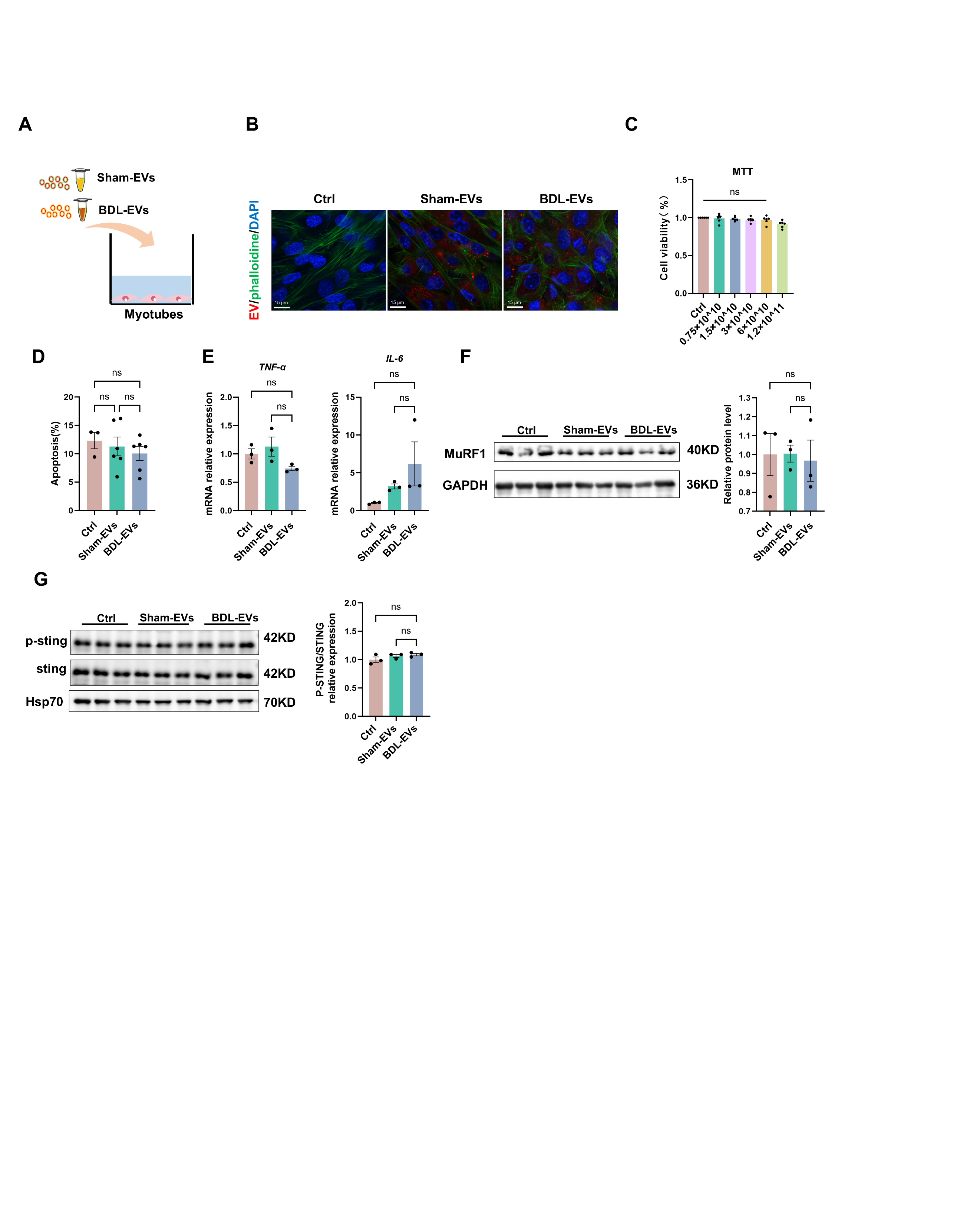
**

**Figure S9 Effect of BDL-EVs on proinflammatory effects in C2C12-derived myotubes cells.** (A) Experimental scheme. Detection of hepatic EVs on myotubes. (B) Representative images of PKH26-labeled EV (red) uptake in C2C12-derived myotubes stained with FITC-phalloidin (green) and DAPI (blue) (scale bar = 15 μm). (C) Cell viability was determined by MTT assay. (n = 6). (D) Determination of apoptosis (%) in C2C12-derived myotubes under hepatic-EVs conditions of different groups. (n = 3/6/6). (E) Expression levels of *IL-6* and *TNF-α* measured by qPCR. (n = 3). (F) Representative blots and quantiﬁed data of the expression of MuRF1 protein. (n = 3). (G) Representative blots and quantiﬁed data of the expression of p-STING and STING proteins in myotubes. (n = 3). ^ns^p ≥ 0.05.


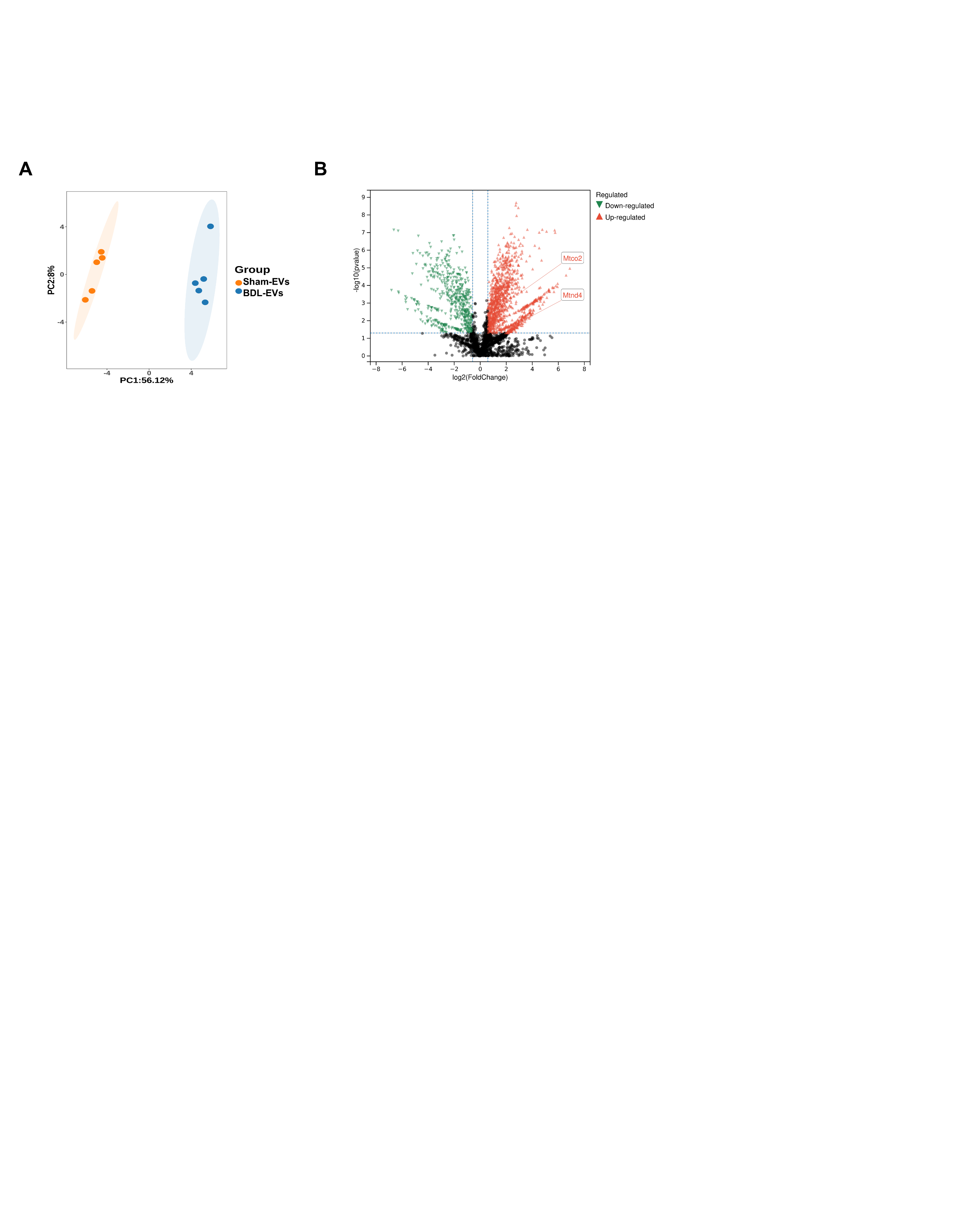


**Figure S10** **LC-MS/MS-based proteomics analysis of Sham-EVs and BDL-EVs.**

(A) PCA scatterplot based on RNA-seq data of Sham-EVs treated with or with BDL-EVs. (n = 5). (B) Volcano plots showing the DEPs (FC > 1.5 and P- adjusted < 0.05) between the Sham-EVs group and the BDL-EVs group. (n = 5).

**
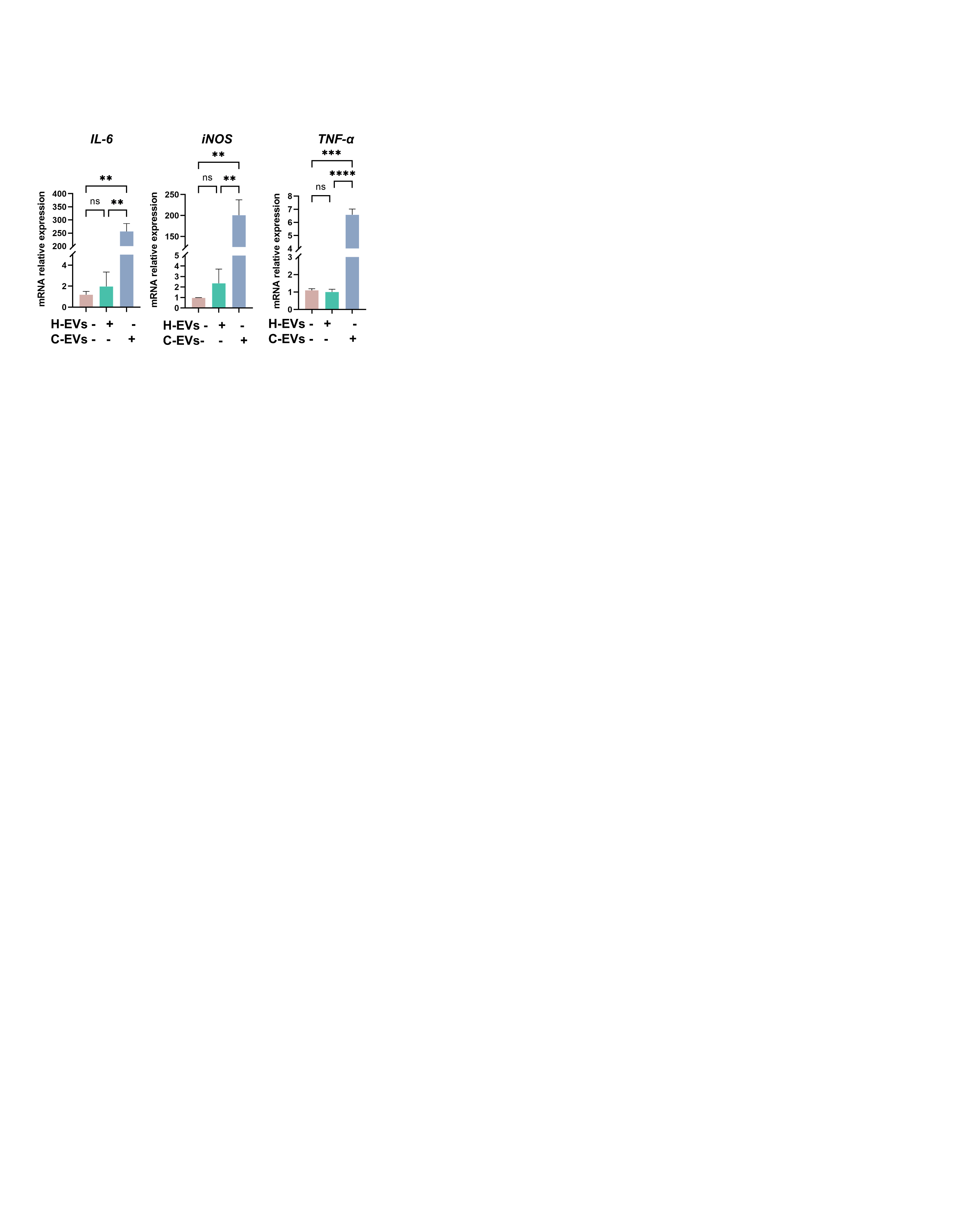
**

**Figure S11** Expression levels of *IL-6*, *iNOS* and *TNF-α* measured by qPCR in macrophages of different groups (n = 3-4). ^ns^p ≥ 0.05, **p < 0.01, ***p < 0.001, ****p < 0.0001.

**
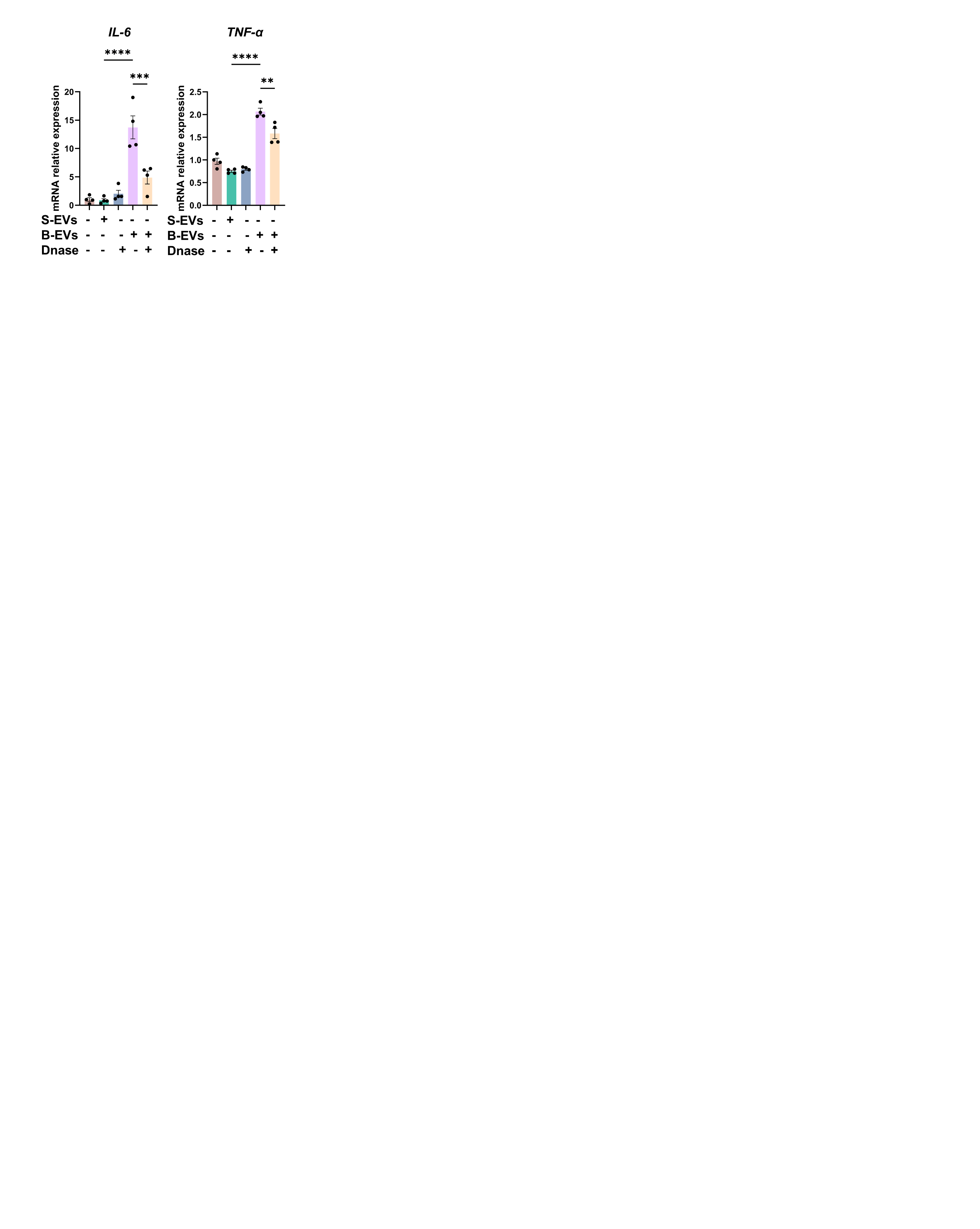
**

**Figure S12** Expression levels of *IL-6* and *TNF-α* measured by qPCR in macrophages of different groups (n = 4). **p < 0.01, ***p < 0.001, ****p < 0.0001.


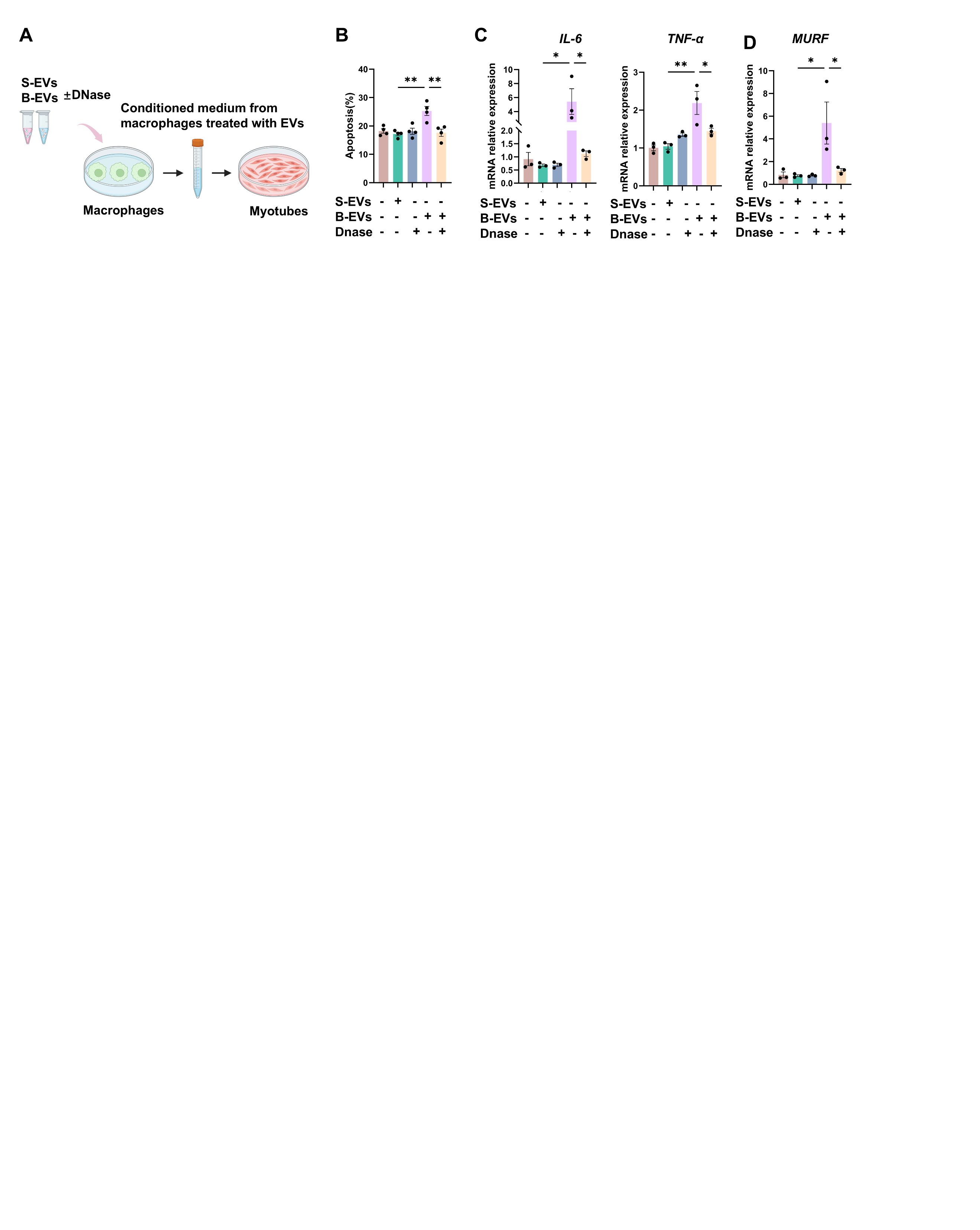


**Figure S13 Effects of macrophages with or without mtDNA depletion on C2C12-derived myotubes.** (A) Experimental scheme. (B) Determination of apoptosis rates (%) in myotubes (n = 4). (C-D) Expression levels of *IL-6*, *TNF-α* and *MURF* in myotubes from different groups were measured via qPCR (n = 3). *p < 0.05, **p < 0.01

**
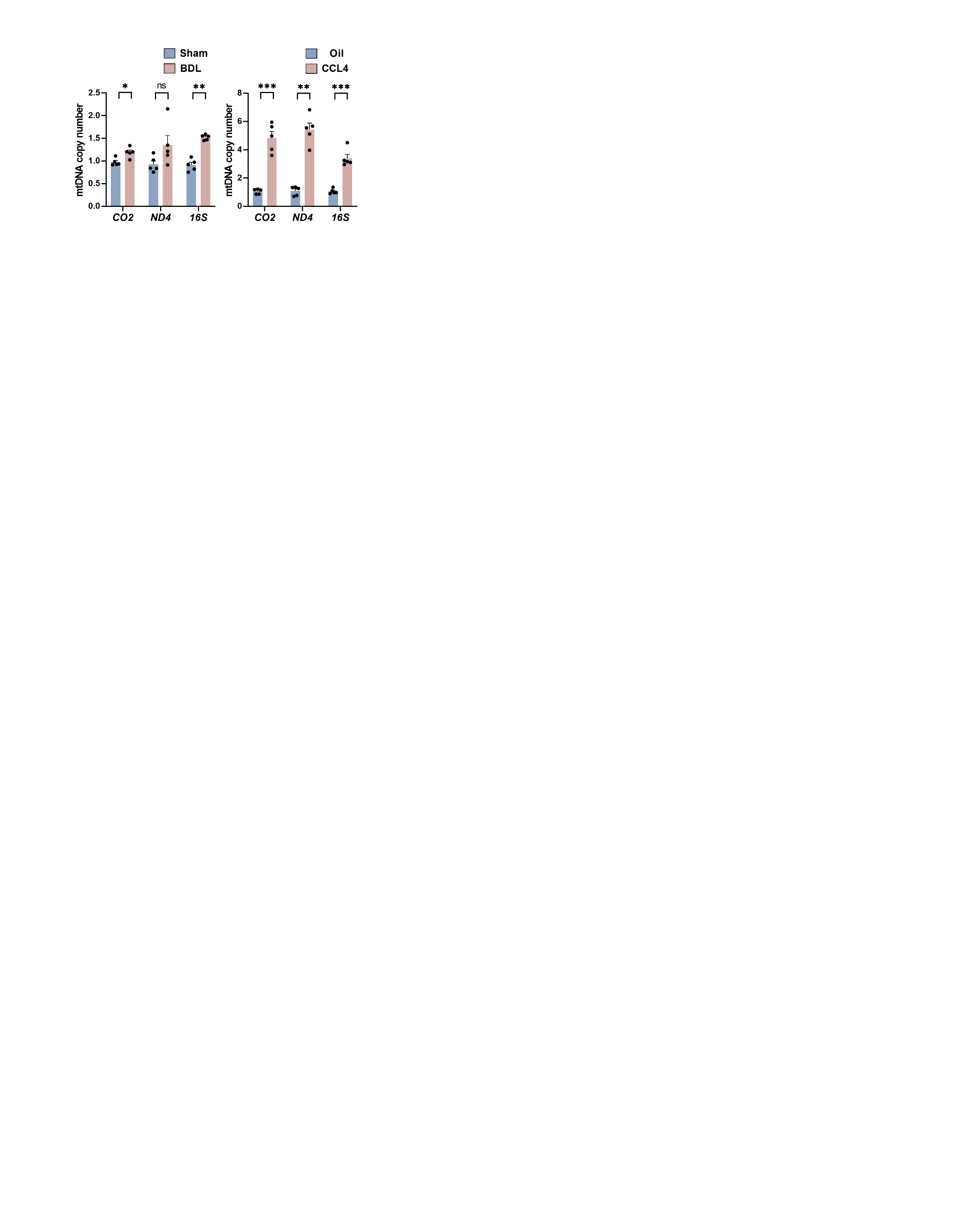
**

**Figure S14 Detecting mtDNA content levels in livers of cirrhosis models.**

(A) Expression levels of *CO2*, *ND4*, *16S* measured by qPCR in Sham and BDL mice. (n = 5). (B) Expression levels of *CO2*, *ND4*, *16S* measured by qPCR in Oil and CCL_4_ mice (n = 5). ^ns^p ≥ 0.05, *p < 0.05, **p < 0.01.

**
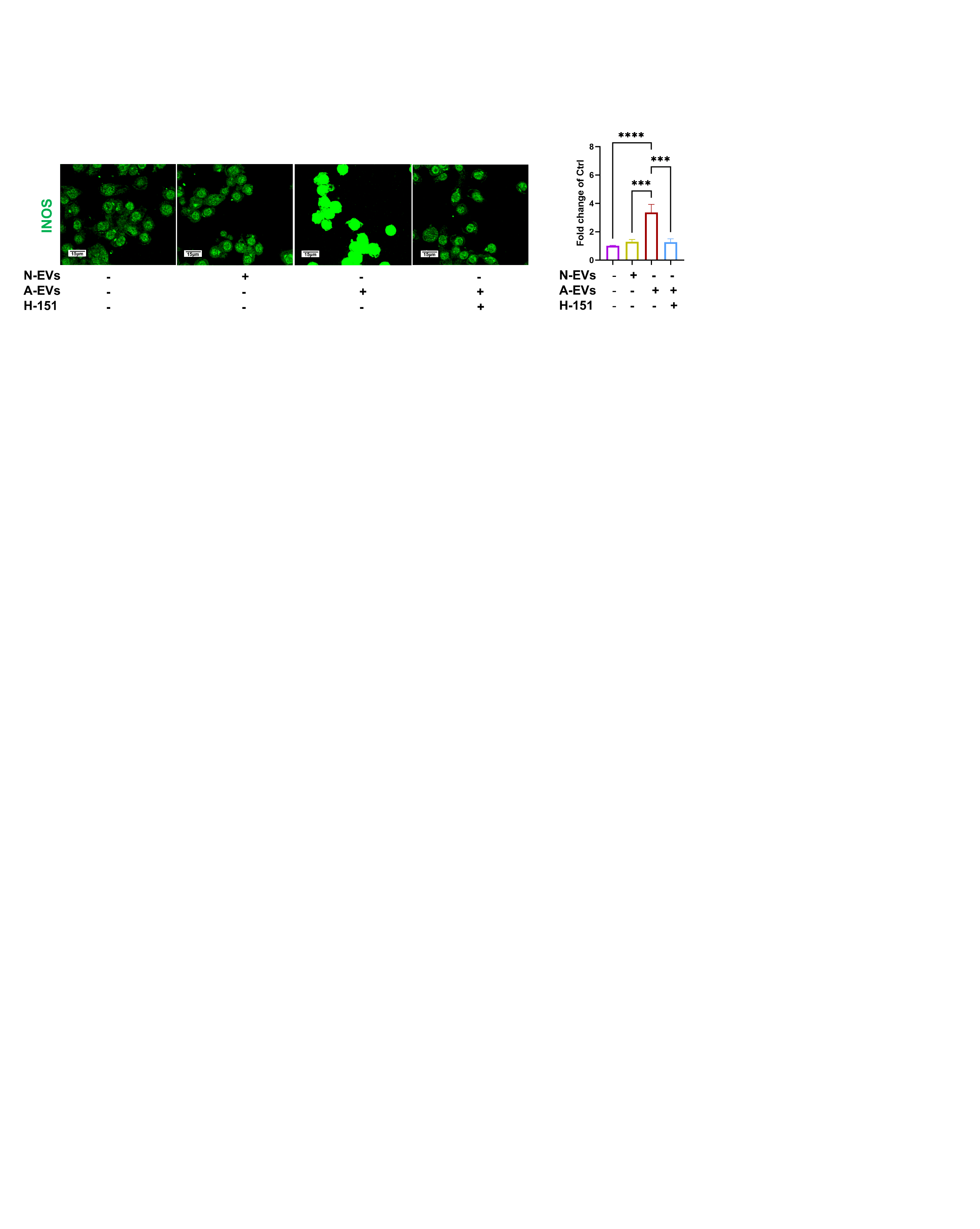
**

**Figure S15 Effect of H-151 and EV treatments on proinflammatory effects and expression of ISGs in macrophages.** Representative image of iNOS staining (scale bar = 15 um, n = 3) and the quantification analysis in different groups. ***p < 0.001, ****p < 0.0001.


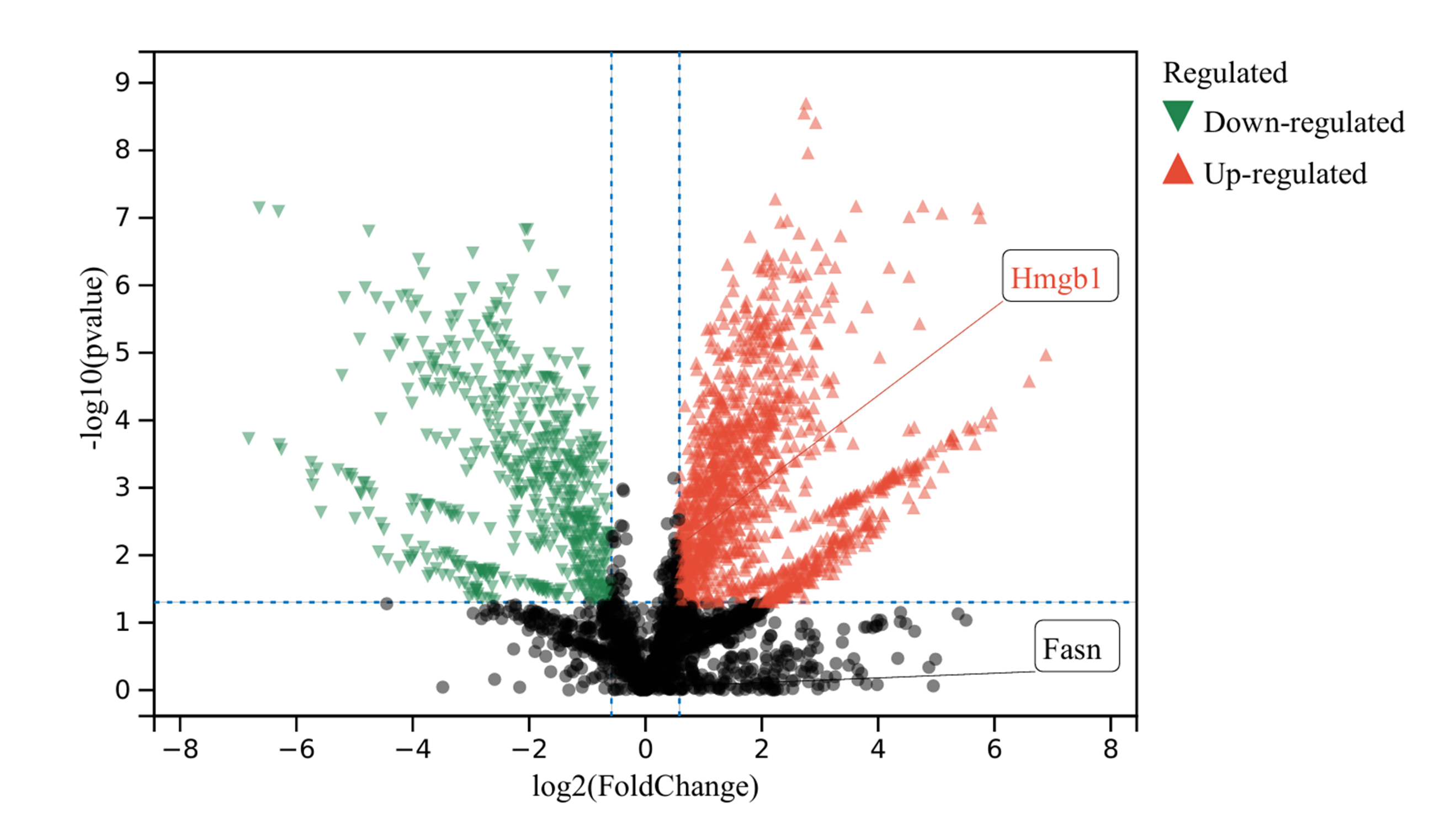


**Figure S16 Expression of Hmgb1 and Fasn in the LC-MS/MS-based proteomics analysis of Sham-EVs and BDL-EVs.** Volcano plots showing the DEPs (FC > 1.5 and P- adjusted < 0.05) between the Sham-EVs group and the BDL-EVs group. (n = 5).

**Table S1** Primers used in RT-qPCR analysis (Mouse)

| Gene |  | Sequence 5 ' - 3 ' | | |  |  |
| --- | --- | --- | --- | --- | --- | --- |
| MuRF1  IL-6 | CAGAGGCAGTTGGATCTCTATG  TGAGGCAGAGTCTCTCTATGT  CTGCAAGAGACTTCCATCCAG  AGTGGTATAGACAGGTCTGTTGG | | | | |  |
| iNOS |  | TCTAGTGAAGCAAAGCCCAACA  CCTCACATACTGTGGACGGG | | | |  |
| TNF-α |  | CTGAGTTCTGCAAAGGGAGAG  CCTCAGGGAAGAATCTGGAAAG |  |  |  |  |
| IL-1β |  | AAGCTTCCTTGTGCAAGTGT  TAGCCCTCCATTCCTGAAAGC |  |  |  |  |
| Actin  CXCL10 |  | GCAGGAGTACGATGAGTCCG  ACGCAGCTCAGTAACAGTCC  CAGGCTCGTCAGTTCTAAGTTTACC  CCTTGGGAAGATGGTGGTTAAGTTC |  |  |  |  |
| ISG15 |  | GCAAGCAGCCAGAAGCAGAC  CGGACACCAGGAAATCGTTACC |  |  |  |  |
| OASL2  STAT2  GAPDH  Rab27a  Rab27b  Smpd3  TFAM  PGC-1α  NDUFS8  ATP5a1 |  | CTTGTGCGGAGGATCAGGTACTAC  CGGACTCAAGCAGCCAGACTAG  ACCCATTCCGCTGTTCGCTATC  CAGTGCCGCTCCTTCCAGTG  GGAGAAACCTGCCAAGTATGA  TCCTCAGTGTAGCCCAAGA  AGTTGATGGAGCGAACTGCT  CCCTACACCAGAGTCTCCCA  GGCAGCATCAGTATCGGCTC  TGATGGTCTTGGTCGGTACCTC  CTCCTCAGAGGTTTCCGGTG  CGGTTGCTTTGGTGACACAG  CACCCAGATGCAAAACTTTCAG  CTGCTCTTTATACTTGCTCACAG  CACCAAACCCACAGAAAACAG  GGGTCAGAGGAAGAGATAAAGTTG  GTTCATAGGGTCAGAGGTCAAG  TCCATTAAGATGTCCTGTGCG  CATTGGTGATGGTATTGCGC  TCCCAAACACGACAACTCC |  |  |  |  |

**Table S2** Primers used in RT-qPCR analysis (Human)

| **Gene** |  | **Sequence 5 ' - 3 '** | | |  |  |
| --- | --- | --- | --- | --- | --- | --- |
| Rab27a  Rab27b | AGTTGATGGAGCGAACTGCT CCCTACACCAGAGTCTCCCA  GGCAGCATCAGTATCGGCTC  TGATGGTCTTGGTCGGTACCTC | | | | |  |
| Smpd3 |  | CTCCTCAGAGGTTTCCGGTG CGGTTGCTTTGGTGACACAG | | | |  |
| Actin |  | TGAAAACAACGTTCTGTCCCCC  AGCCCAGACGGAAACCGTAG |  |  |  |  |
| TFAM |  | GGTGCAGACAGGACAGTGTT  CCCTACTCCCAGACTGCTCT |  |  |  |  |
| PGC-1α  NDUFS8 |  | CCCCATGGATGAAGGGTACTT  GGGGAGGTCTCATCCATTGC  GAACCGGCCACCATCAACTA  AAGCCGCAGTAGATGCACTT |  |  |  |  |
| ATP5a1  RAB11A |  | ACTGCAAAGATGCTGTCCGT  AGTAAGTGCGGCGTAGACTG  TGCAGAAAAGAATGAAGCAAATGT  TTCTGACAGCACTGCACCTT |  |  |  |  |
| RAB35 |  | CGCCAAGGAGAATGTCAACG |  |  |  |  |
|  |  | AACGGCACGAAACTGAGACT |  |  |  |  |
| Rab7A |  | GGTTCCAGTCTCTCGGTGTG |  |  |  |  |
|  |  | TTGAATGTGTTGGGGGCAGT |  |  |  |  |

**Table S3** Clinical characteristics of patients with cirrhosis with or without sarcopenia

| Parameters | Cirrhosis without sarcopenia  (N=82) | Cirrhosis with sarcopenia  (N=55) | P value | |  |
| --- | --- | --- | --- | --- | --- |
| Age (years) | 56.0 (49.0, 63.0) | 61.0 (52.0,67.0) |  | .068 | |
| Sex (M: F) | 16:66 | 4:51 |  | .047 | |
| Decompensation | 21/61 | 20/35 |  | .178 | |
| TBiL (μmol/L) | 23.0 (16.0, 35.4) | 18.7 (16.1,29.7) |  | .177 | |
| ALT (IU/L) | 41.5 (23.8,71.0) | 47.0 (26.0,65.0) |  | .370 | |
| AST (IU/L) | 54.5 (33.5,79.3) | 61.0 (39.0, 96.0) |  | .404 | |
| ALB (g/L) | 39.0 (34.0,43.1) | 39.4 (34.4, 44.9) |  | .680 | |
| GLB(g/L) | 34.3(30.4, 39.5) | 33.6(30.7, 39.5) |  | .716 | |
| ALP (IU/L) | 168.5(121.8, 276.0) | 229.0(137.0, 316.0) |  | .134 | |
| GGT (IU/L) | 81.5(38.8, 277.3) | 147.0(55.0, 284.0) |  | .053 | |
| SMA (cm^2^) | 118.7(107.6, 133.4) | 94.5(88.3, 100.8) |  | <.001 | |
| SMI | 42.3 (38.7, 46.7) | 32.6 (31.0, 34.5) |  | <.001 | |

Normal range: TBIL: 5–28 μmol/L; ALT: male: 50 IU/L, female: 40 IU/L; AST: male: 40 IU/L, female: 35 IU/L; ALP: male: 51–160, female: < 50 years old: 35–100 IU/L, ≥ 50 years old: 50-135 IU/L; GGT: male: 60 IU/L, female: 45 IU/L; ALB: 40-55 g/L; GLB: 20-40 g/L; TBIL, total bilirubin; ALT, alanine aminotransferase; AST, aspartate aminotransferase; ALB, albumin; GLB, globulin; ALP: alkaline phosphatase; GGT, gamma-glutamyl transferase; SMA, skeletal muscle area; SMI, skeletal muscle index. The data are presented as medians with interquartile ranges for nonnormally distributed variables.

**Table S4** Clinical characteristics of patients with cirrhosis underwent TIPS.

| Parameters |  |  | | | Amount (n=22) | |
| --- | --- | --- | --- | --- | --- | --- |
| PPG (mmHg) | | | | | | 17.5 (6-31) |
| TBil (umol/L) | | | | | | 19.15 (2.7-45.6) |
| ALT (IU/L) | | |  |  |  | 22 (4-126) |
| AST (IU/L) |  |  |  |  |  | 28 (14-116) |
| Albumin (g/L) | | |  |  |  | 32.2 (23.6-46.4) |

Data are shown as median and ranges for continuous variables. PPG, portosystemic pressure gradient; TBiL, total bilirubin; AST, aspartate transaminase; ALT, alanine transaminase. The data were presented as medians with interquartile ranges for nonnormally distributed variables.

**Table S5** Primers used in mtDNA copy number assay

| **Gene** |  | **Sequence 5 ' - 3 '** | | |  | **Species** |
| --- | --- | --- | --- | --- | --- | --- |
| CO2 | ATAACCGAGTCGTTCTGCCAAT  TTTCAGAGCATTGGCCATAGAA | | | | |  |
| Rps18 |  | TGTGTTAGGGGACTGGTGGACA  CATCACCCACTTACCCCCAAAA | | | |  |
| mtND4  16S |  | TAATCGCACATGGCCTCACA  GAAGTCCTCGGGCCATGATT  TGAACGGCTAAACGAGGGTC  AGCTCCATAGGGTCTTCTCGT |  |  |  | Mouse |
| ND1  B2M |  | CACTTTCCACACAGACATCA  TGGTTAGGCTGGTGTTAGGG  TGTTCCTGCTGGGTAGCTCT  CCTCCATGATGCTGCTTACA |  |  |  |  |
| mtND4 |  | CTACCACTGACATGACTTTCCAAAA |  |  |  | Human |
|  |  | AGGAAAAGGTTGGGGAACAGCTA |  |  |  |  |
| CO2 |  | TCATGAGCTGTCCCCACATTAG  GGGCATGAAACTGTGGTTTGCT |  |  |  |  |

**Table** **S6** Gene profiles of muscles injected with Sham-EVs or muscles injected with BDL-EVs are provided in an excel file.

**Table** **S7** Protein profiles of Sham-EVs and BDL-EVs are provided in an excel file.
